# Multi-omics profiling and experimental verification of tertiary lymphoid structure-related genes: molecular subgroups, immune infiltration, and prognostic implications in lung adenocarcinoma

**DOI:** 10.3389/fimmu.2024.1453220

**Published:** 2024-09-19

**Authors:** Sixuan Wu, Junfan Pan, Qihong Pan, Lijun Zeng, Renji Liang, Yuehua Li

**Affiliations:** ^1^ Department of Oncology, The First Affiliated Hospital, Hengyang Medical School, University of South China, Hengyang, Hunan, China; ^2^ Clinical Oncology School of Fujian Medical University, Fujian Cancer Hospital, Fuzhou, China; ^3^ Department of Thoracic Surgery, The First Affiliated Hospital, Hengyang Medical School, University of South China, Hengyang, China

**Keywords:** lung adenocarcinoma, tertiary lymphoid structures, tumor microenvironment, tumor mutation burden, overall survival

## Abstract

Lung adenocarcinoma (LUAD), characterized by a low 5-year survival rate, is the most common and aggressive type of lung cancer. Recent studies have shown that tertiary lymphoid structures (TLS), which resemble lymphoid structures, are closely linked to the immune response and tumor prognosis. The functions of the tertiary lymphoid structure-related genes (TLS-RGs) in the tumor microenvironment (TME) are poorly understood. Based on publicly available data, we conducted a comprehensive study of the function of TLS-RGs in LUAD. Initially, we categorized LUAD patients into two TLS and two gene subtypes. Subsequently, risk scores were calculated, and prognostic models were constructed using seven genes (CIITA, FCRL2, GBP1, BIRC3, SCGB1A1, CLDN18, and S100P). To enhance the clinical application of TLS scores, we have developed a precise nomogram. Furthermore, drug sensitivity, tumor mutational burden (TMB), and the cancer stem cell (CSC) index were found to be substantially correlated with the TLS scores. Single-cell sequencing results reflected the distribution of TLS-RGs in cells. Finally, we took the intersection of overall survival (OS), disease-specific survival (DSS), and progression-free interval (PFI) prognosis-related genes and then further validated the expression of these genes by qRT-PCR. Our in-depth investigation of TLS-RGs in LUAD revealed their possible contributions to the clinicopathological features, prognosis, and characteristics of TME. These findings underscore the potential of TLS-RGs as prognostic biomarkers and therapeutic targets for LUAD, thereby paving the way for personalized treatment strategies.

## Introduction

Lung cancer is the predominant form of cancer and the leading cause of cancer-related deaths worldwide (1–4). Approximately 80–85% of these cases are non-small cell lung cancer (NSCLC) (5). Lung adenocarcinoma (LUAD) is the most prevalent subtype of NSCLC (6, 7). The five-year survival rate for LUAD is approximately 15% because most patients are diagnosed at an advanced stage (8). Immunotherapy significantly alters the treatment course for cancer patients (9–11). Specifically, therapeutic strategies for metastatic NSCLC, either in a first- or second-line setting, have resulted in unprecedentedly prolonged survival in some patients (12, 13). Nevertheless, not every patient responds to immunotherapy, and only a small fraction achieve long-term survival (14). Therefore, the identification of additional biomarkers is essential to enhance the efficacy of precision immunotherapy in NSCLC patients.

Tertiary lymphoid structures (TLS) are ectopic formations of lymphoid tissue acquired from inflammatory, infectious, or tumoral tissues (15, 16). TLS include a T cell zone containing mature dendritic cells and a germinal center containing proliferating B cells, follicular dendritic cells, and high endothelial venules (17–20). Emerging as a significant predictor of patient outcomes, the TLS has been identified in the pathological evaluation of numerous cancers (21, 22). Due to their distinct immunogenic niches, they represent excellent candidates for enhancing therapeutic efficacy and for predicting and assessing the effectiveness of immunotherapy drugs (21, 23, 24). Specific studies have examined the prognostic significance of TLS in a range of tumor types, including pancreatic ductal adenocarcinomas (25, 26), esophageal squamous cell carcinoma (27), breast cancer (28, 29), endometrial cancer (30, 31), cholangiocarcinoma (32, 33), gastric carcinoma (34, 35), human melanoma (36), renal cell cancer (37), and hepatocellular carcinoma (38), among others. These studies showed that TLS affects patient prognosis and influences immunological infiltration, thereby enhancing patient survival. Therefore, a comprehensive evaluation of TLS in LUAD is crucial, particularly focusing on changes in tertiary lymphoid structure-related genes (TLS-RGs). Identifying different TLS-RG subgroups among LUAD patients may potentially improve their prognosis.

Patients with LUAD were initially divided into two distinct subgroups based on TLS-RGs expression levels. Following the identification of differentially expressed genes (DEGs) based on the two TLS subtypes, patients were categorized into two distinct gene subtypes. We created a scoring method to assess the immunological landscape and predict prognosis. Additionally, we investigated how TLS-RGs influence the tumor microenvironment (TME), cancer stem cells (CSC), tumor mutational burden (TMB), and drug sensitivity in LUAD. Moreover, we analyzed TLS-RGs through single-cell sequencing to offer a comprehensive description of their prognostic significance. Finally, the prognosis-related genes were verified by qRT-PCR. Specifically, acquiring a more profound understanding of the role played by TLS-RGs not only facilitates a comprehensive exploration of TLS as potential therapeutic targets for treating LUAD but also contributes to improving the prognosis of LUAD patients through informed treatment strategies and personalized interventions.

## Materials and methods

### LUAD dataset download and TLS-RGs acquisition

A process map outlining the current study was depicted in **Supplementary Figure 1**. TCGA database provides RNA expression, somatic mutation data, and clinical characteristics for LUAD. The TCGA database can be accessed at https://portal.gdc.cancer.gov/. A suitable number of samples and comprehensive clinical information were obtained from the Gene Expression Omnibus (GEO) database, accessible at https://www.ncbi.nlm.nih.gov/geo/. From this database, the LUAD-related dataset GSE13213 was located and downloaded. The FPKM values of 541 LUAD patients and 59 normal patients from the TCGA database were converted to TPM values and normalized. Subsequently, the GSE13213 data (117 LUAD patients) was merged to create a comprehensive expression matrix. These datasets were then systematically organized and processed utilizing Strawberry Perl (version 5.30.0.1). Immunohistochemical images of lung cancer tissues and their corresponding normal tissues were obtained from the Human Protein Atlas (HPA) database to assess the protein expression levels of 5 TLS prognostic genes. Previous studies provided 39 TLS-RGs (39). Gene details are available in **Supplementary Table 1**.

LUAD tissues and corresponding paracarcinoma tissue samples were collected from 30 lung cancer surgery patients at Fujian Cancer Hospital. This study was approved by the hospital’s Ethics Committee (number: K2023-417-01). Informed consent was obtained from all participants prior to enrollment.

### Analysis of TLS-RGs using consensus clustering

We utilized the “ConsensusClusterPlus” tool in the R package to conduct consensus unsupervised clustering analysis. The following standards were used to produce this clustering: initially, the curve representing the cumulative distribution function (CDF) increased steadily and gradually. None of the groups had small sample sizes. Finally, there was an increase in the intragroup correlations and a decrease in the intergroup correlations following clustering. The classification of several subgroups can be evaluated using principal component analysis (PCA), which partially reflects the variations among subgroups. Kaplan-Meier (KM) curves were also generated to illustrate differences in survival rates between different subgroups.

### DEGs identification and functional enrichment analysis

229 DEGs between the TLS subtypes were determined utilizing the R “limma” package. The p < 0.05 and a fold change of 2.0 were used as the criteria. The molecular signaling pathways involved were determined using the Kyoto Encyclopedia of Genes and Genomes (KEGG) enrichment analysis (40). Gene ontology (GO) enrichment analysis facilitated the classification and description of gene and protein activities (41). Differential analysis at the signaling system level was performed using gene-set variation analysis (GSVA) (42).

### Creation of TLS scores

First, univariate Cox regression analysis was conducted on the dataset to identify the DEGs associated with LUAD overall survival (OS). Secondly, based on the expression levels of prognostic TLS-RGs, patients were stratified into two subtypes (TLS gene subtypes A and B) for further investigation using an unsupervised clustering method. Finally, a 1:1 randomization process was employed to divide all patients with LUAD into training and test sets. The test set and the training set each contained 312 patients. The training set was utilized to create TLS-related prognostic scores. To sum up, we utilized the “glmnet” R package alongside Lasso Cox regression to reduce overfitting risk linked to TLS-related prognostic genes. Through multivariate Cox regression analysis, candidate genes were identified, and predictive TLS scores were subsequently derived within the training set.

Patients within the training set were stratified into two groups: those categorized as low-risk (TLS scores above median) and those identified as high-risk (TLS scores below median). In a similar manner, the testing group was divided into low-risk and high-risk groups. Receiver operating characteristic (ROC) curves were generated, and KM survival analyses were performed for each group.

### Cell culture for qRT-PCR analysis

The cell lines Beas-2B, PC9, A549, H1299, and HCC827 used in our study were purchased from procell (Wuhan, China). The frozen stocks of Beas-2B, PC9, A549, H1299, and HCC827 cells were thawed in a 37°C water bath. Subsequently, the cells were cultured in 10 cm dishes with RPMI-1640 medium supplemented with 10% fetal bovine serum and 1% penicillin/streptomycin, and then incubated in a 37°C humidified atmosphere with 5% carbon dioxide. The TRIzol reagent (Invitrogen, Carlsbad, CA, USA) was used to extract total RNA. Total RNA and the PrimeScript RT Reagent Kit (Takara) were used to create complementary DNA. Takara SYBR Green assays were utilized for qRT-PCR analysis. The 2-ΔΔCt approach was used to compile qRT-qPCR data normalized using GAPDH. The primer sequences used for the qRT-PCR are listed in **Supplementary Table 3**.

### Assessment of immune cells infiltration, and TME

The ESTIMATE algorithm was utilized to assess the stromal scores and immune scores of each patient. CIBERSORT was utilized to measure the number of infiltrating immune cells in heterogeneous samples from both the low- and high-risk groups, aiming to assess the percentage of tumor-infiltrating immune cells (TIICs) in the TME. Seven genes with TLS scores were compared with the fractions of the 19 infiltrating immune cells.

### Assessment of TMB, CSC, and mutation

The R package “maftools” (43) was utilized to create the mutation annotation format. This facilitated the comparison of somatic mutations among LUAD patients. Additionally, we investigated the correlation between CSC, TMB, and the two risk groups.

### Drug susceptibility analysis

We utilized the “pRRophetic” software to compute the semi-inhibitory concentration (IC50) values of medications commonly used for treating LUAD. This analysis aimed to examine variations in the therapeutic responses of these treatments among patients belonging to the two groups.

### Establishment and verification of the nomogram

Utilizing findings from independent prognostic studies, we effectively utilized clinical characteristics and risk scores to construct a predictive nomogram, facilitated by the “rms” program. A comprehensive assessment of the nomogram’s performance ensued. Additionally, a calibration curve was constructed to assess the predictive accuracy of the nomogram.

### Data processing for single-cell sequencing

The GEO database provided the NSCLC scRNA-seq datasets GSE143423, GSE146100, and GSE153935. Utilizing the R package “Seurat” (44), the samples were combined. Cell data satisfying the specified criteria were preserved, including gene counts ranging from 300 to 7,000 and total transcript count under 100,000. Conversely, cell data with gene counts of three or fewer cells and those with fewer than 300 genes detected in a single cell were filtered out. During the manual annotation of various cell clusters, auxiliary annotations were obtained from the CellMarker database, the R package “singleR,” and relevant references.

### Statistical analysis

In this study, we employed R software (version 4.3.2) and GraphPad Prism 9 for data processing, analysis, and visualization. Quantitative variables were analyzed using independent samples t-tests. The effectiveness of R software in predicting survival outcomes was evaluated through ROC curve analysis and KM survival analysis. Statistical significance was set at P < 0.05 for comparisons between groups.

## Results

### Transcriptional and genetic changes of TLS-RGs in LUAD

**Figure 1:**
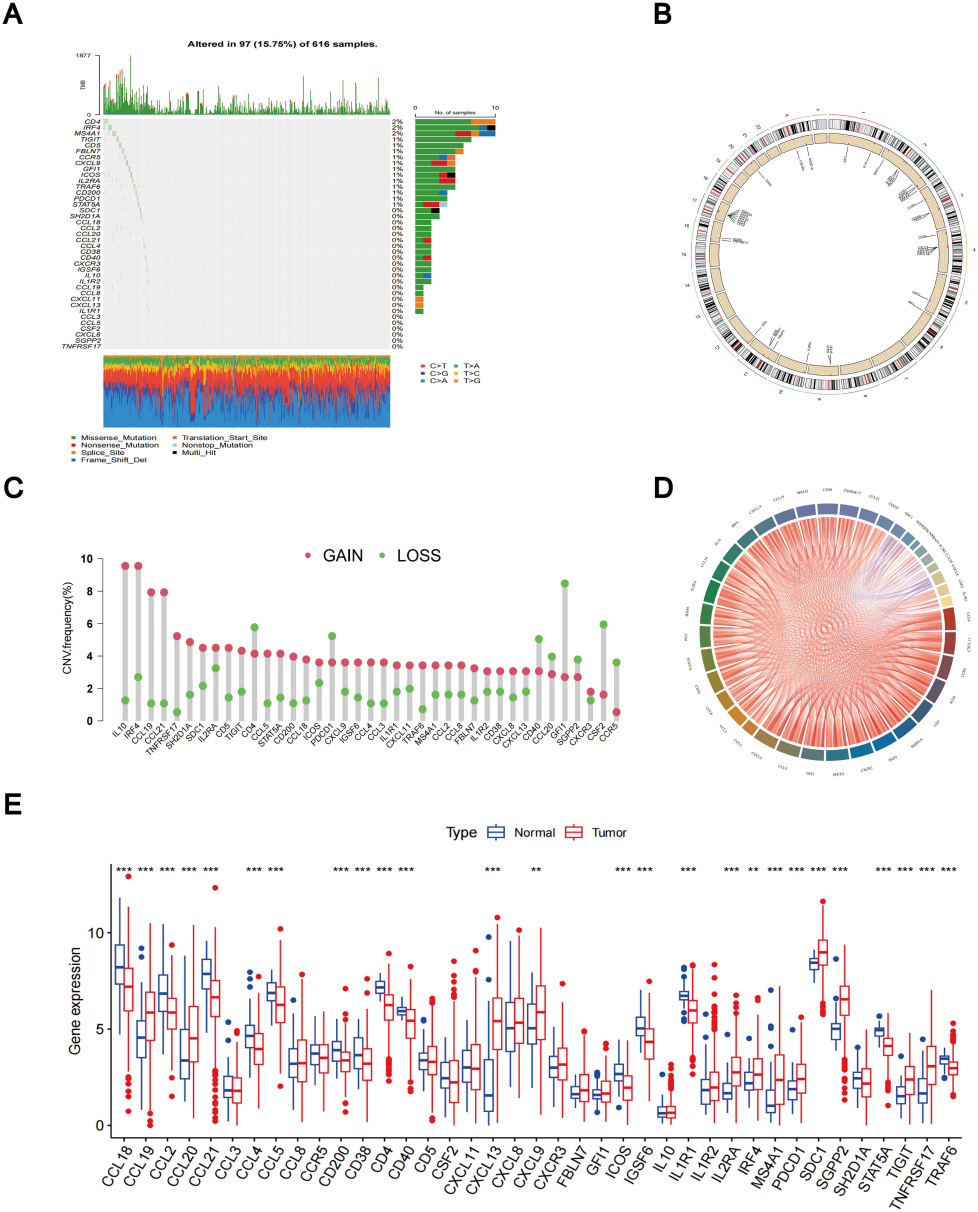
Different mutations, CNV, and expression of TLS-RGs in TCGA cohort. **(A)** The somatic mutation frequency of TLS-RGs. **(B)** The location of CNV alterations of TLS-RGs on 23 chromosomes. **(C)** The CNV frequency of TLS-RGs in LUAD. **(D)** Correlation analysis for TLS-RGs. **(E)** Expression of TLS-RGs in both normal and LUAD tissues. **p < 0.01; ***p < 0.001.

According to the TCGA dataset, a total of 39 TLS-RGs were identified. A summary analysis of the occurrence of somatic mutations in the 39 TLS genes showed that TLS mutations were present in 97 (15.75%) of the 616 LUAD samples (**Figure 1A**). The most frequent mutations were in CD4 (2%), IRF4 (2%), and MS4A1 (2%). The locations of the copy number variation (CNV) changes in the TLS-RGs on the corresponding chromosomes are displayed in **Figure 1B**. In the copy number circle diagram, the outermost circle represents the chromosome, with corresponding chromosome positions labeled as TLS-RGs. As depicted in **Figure 1B**, red labeling signifies genes with a higher frequency of CNV increase, while blue labeling indicates genes with a higher frequency of CNV deletion. Subsequently, we showed that the levels of IL10, IRF4, CCL19, and CCL21 were generally elevated in CNV, whereas the levels of GFI1 and CSF2 levels were decreased (**Figure 1C**). At the same time, we performed a correlation analysis of TLS-RGs (**Figure 1D**). Additionally, we investigated the differential expression of the 39 TLS-RGs in LUAD tumors and normal tissues. Among the 26 TLS-RGs exhibiting significant expression differences in LUAD, 12 genes displayed up-regulation, while 14 showed down-regulation (**Figure 1E**). Our investigation uncovered noteworthy variances in both TLS-RGs expression levels and genetic profiles between LUAD and control specimens.

The high expression of CCL2, CCL3, CCL5, CCL18, CCL19, CCR5, CD4, CD5, CD40, CSF2, CXCL9, CXCR3, FBLN7, GFI1, ICOS, IGSF6, IL1R1, IL10, IRF4, MS4A1, SDC1, SH2D1A, STAT5A, TIGIT, and TNFRSF17 was associated with improved OS in LUAD patients. In contrast, the elevated expression of CXCL8, IL1R2, CCL20, SGPP2, CXCL11, and CCL21 correlated with poorer OS (**Supplementary Figure 2**).

### Prognostic analysis of TLS-RGs, TLS subtypes confirmation, and immune infiltration analysis

**Figure 2:**
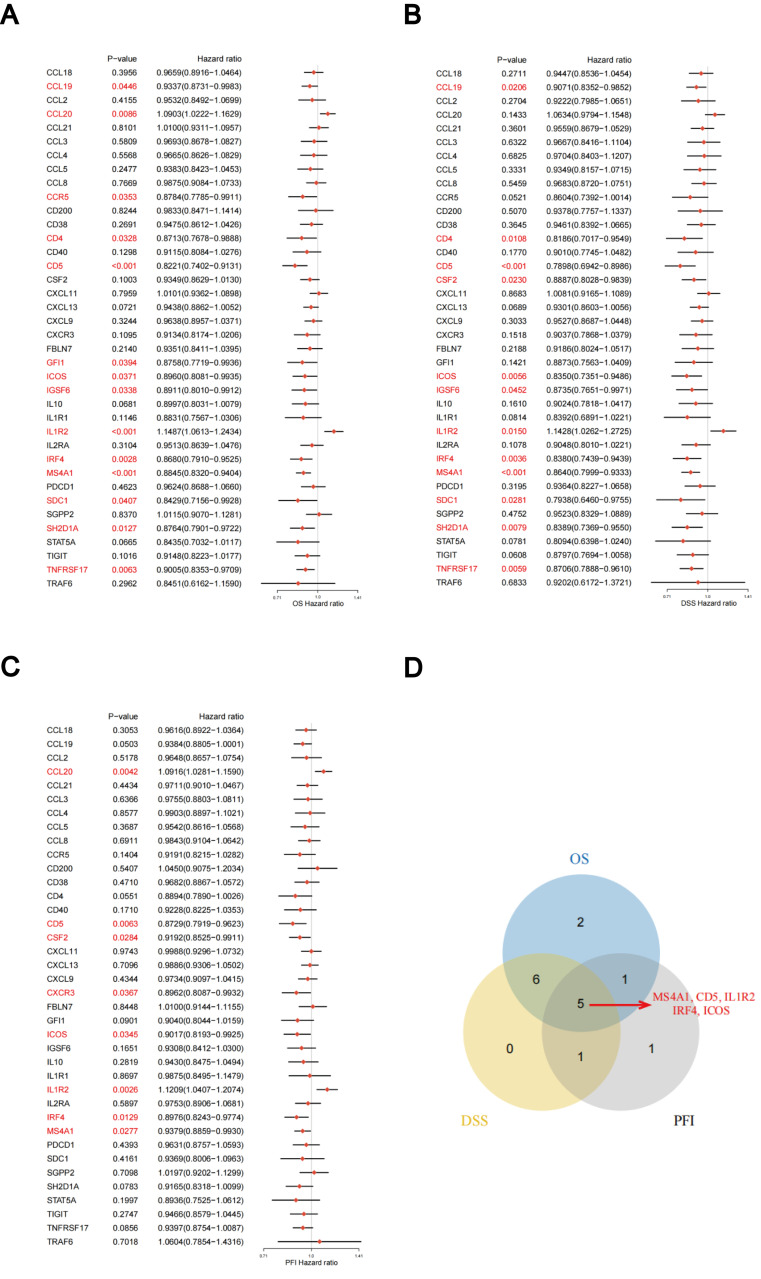
Association between TLS-RGs expression and OS, DSS, and PFI. **(A)** Forest plot of OS relationships in 39 TLS-RGs. **(B)** Forest plot of DSS relationships in 39 TLS-RGs. **(C)** Forest plot of PFI relationships in 39 TLS-RGs. **(D)** Venn diagram of OS, DSS, and PFI prognostic genes.

**Figure 3:**
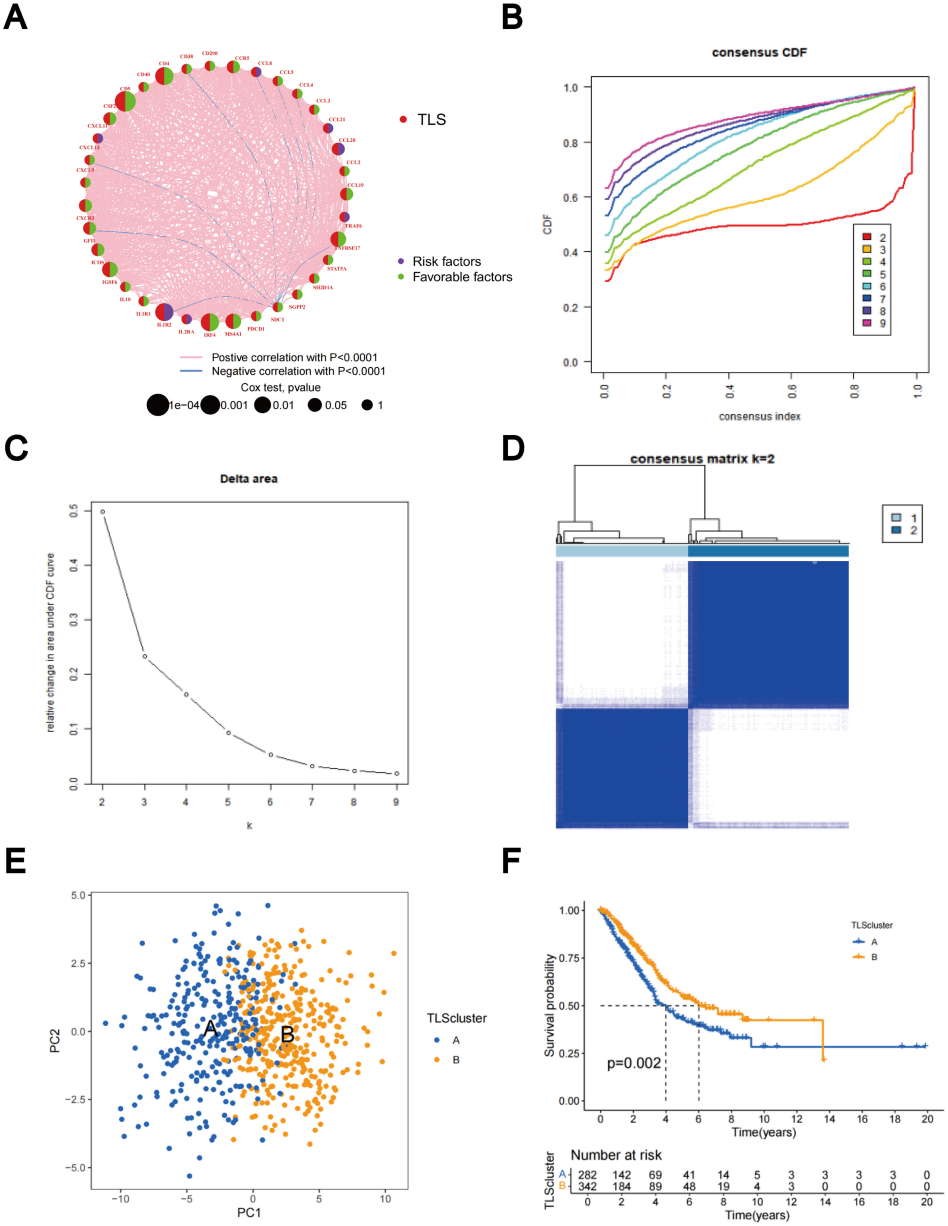
Identification of TLS-RG subgroups in LUAD. **(A)** Prognostic network diagram of TLS-RGs. **(B)** For each tested value of k, the CDF illustrates the cumulative fraction of each sample co-clustering at the specified consensus index, where 1.0 indicates complete co-clustering (100% of the time). **(C)** The consensus clustering delta area curve depicts the variation in the area under the CDF curve concerning k - 1 for each category number k. **(D)** Heatmap of the consensus matrix indicating the correlation region and two clusters (k = 2). **(E)** Significant differences in transcriptomes between the two subtypes are shown by PCA. **(F)** Analysis of KM survival between Clusters A and B.

The predictive significance of the 39 TLS-RGs for OS, disease-specific survival (DSS), and progression-free interval (PFI) in patients with LUAD was determined using univariate Cox regression (**Figures 2A–C**). Concurrently, we examined the intersection of OS, DSS, and PFI prognostic genes (**Figure 2D**). In the prognostic network diagram, nodes depict TLS-RGs, with the left semicircle color indicating gene attributes and the right semicircle representing gene risk, with high-risk genes depicted in purple and low-risk genes in green. Larger nodes denote genes more likely to be prognostically relevant. Lines between nodes signify co-expression relationships (**Figure 3A**). This figure illustrates the interconnections among TLS genes, regulatory linkages, and their significance in predicting the prognosis of LUAD patients. We utilized a consensus clustering algorithm to classify patients according to the expression profiles of the 39 TLS-RGs (**Supplementary Figure 3**). Our findings indicate that the entire cohort could be divided into subtypes A (n = 285) and B (n = 348), with k = 2 appearing to be the best choice (**Figures 3B–D**). PCA uncovered notable distinctions in the transcription profiles of TLS between the two subtypes (**Figure 3E**). According to the KM curves, it was observed that patients categorized as subtype B demonstrated a significantly longer OS compared to those categorized as subtype A (p = 0.002; **Figure 3F**).

**Figure 4:**
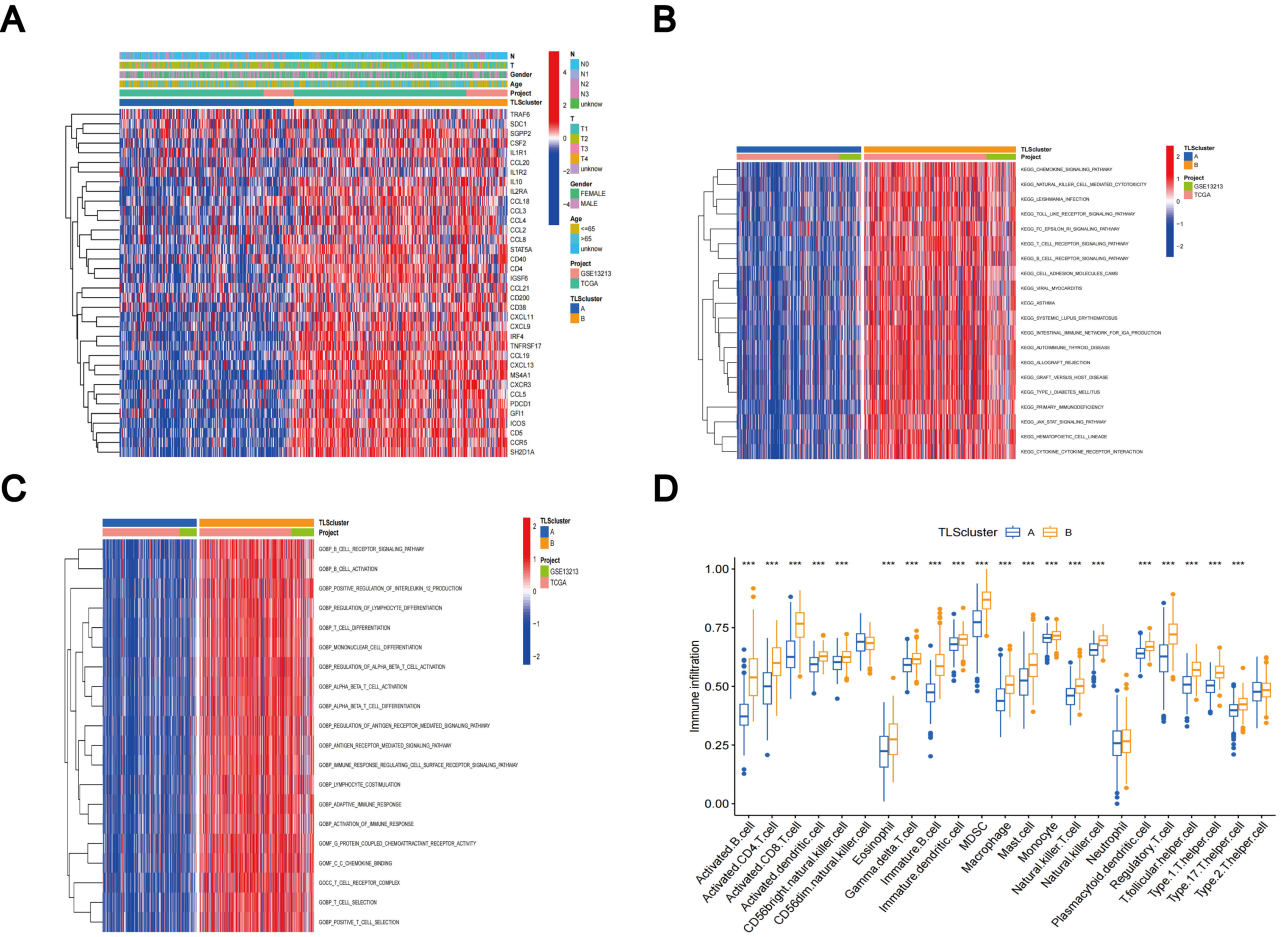
Clinicopathologic features, enrichment analysis, and immune cells infiltration of TLS subtypes. **(A)** Clinicopathological characterization of different TLS subtypes. **(B)** GSVA of biological pathways in the TLS subtypes. **(C)** GSVA of molecular function in the TLS subtypes. **(D)** The characterization of 23 immune cells in the TLS subtypes. ***p < 0.001.

The comparative analysis of clinicopathological features among distinct subtypes of LUAD, conducted using data from both the TCGA and GSE13213 databases, revealed significant distinctions in TLS-RGs expression patterns and clinicopathological attributes. In addition, most TLS-RGs were upregulated in cluster B (**Figure 4A**). GSVA enrichment analysis revealed that subtype B exhibited notable enrichment in fully activated immune pathways. This included the activation of various pathways such as the chemokine signaling pathway, natural T and B cell receptor signaling pathways, cytokine receptor interactions, as well as Toll-like and Jak-stat receptor signaling pathways (**Figure 4B**). GSVA analysis also revealed enrichment of molecular functions in B cell activation, T cell differentiation, lymphocyte co-stimulation, and immune response activation (**Figure 4C**). Using the CIBERSORT algorithm, we evaluated the correlation between the two TLS subtypes and the 23 human immune cell subpopulations in each LUAD sample, aiming to further understand the function of TLS-RGs within the LUAD TME. Our analysis revealed a notable discrepancy in the infiltration levels of most immune cells between the two subtypes (**Figure 4D**). Specifically, subtype B exhibited elevated levels of 20 immune cell types compared to subtype A, including activated B cells, activated CD4+ T cells, macrophages, and activated CD8+ T cells (**Figure 4D**).

### Gene subtypes identification based on DEGs

**Figure 5:**
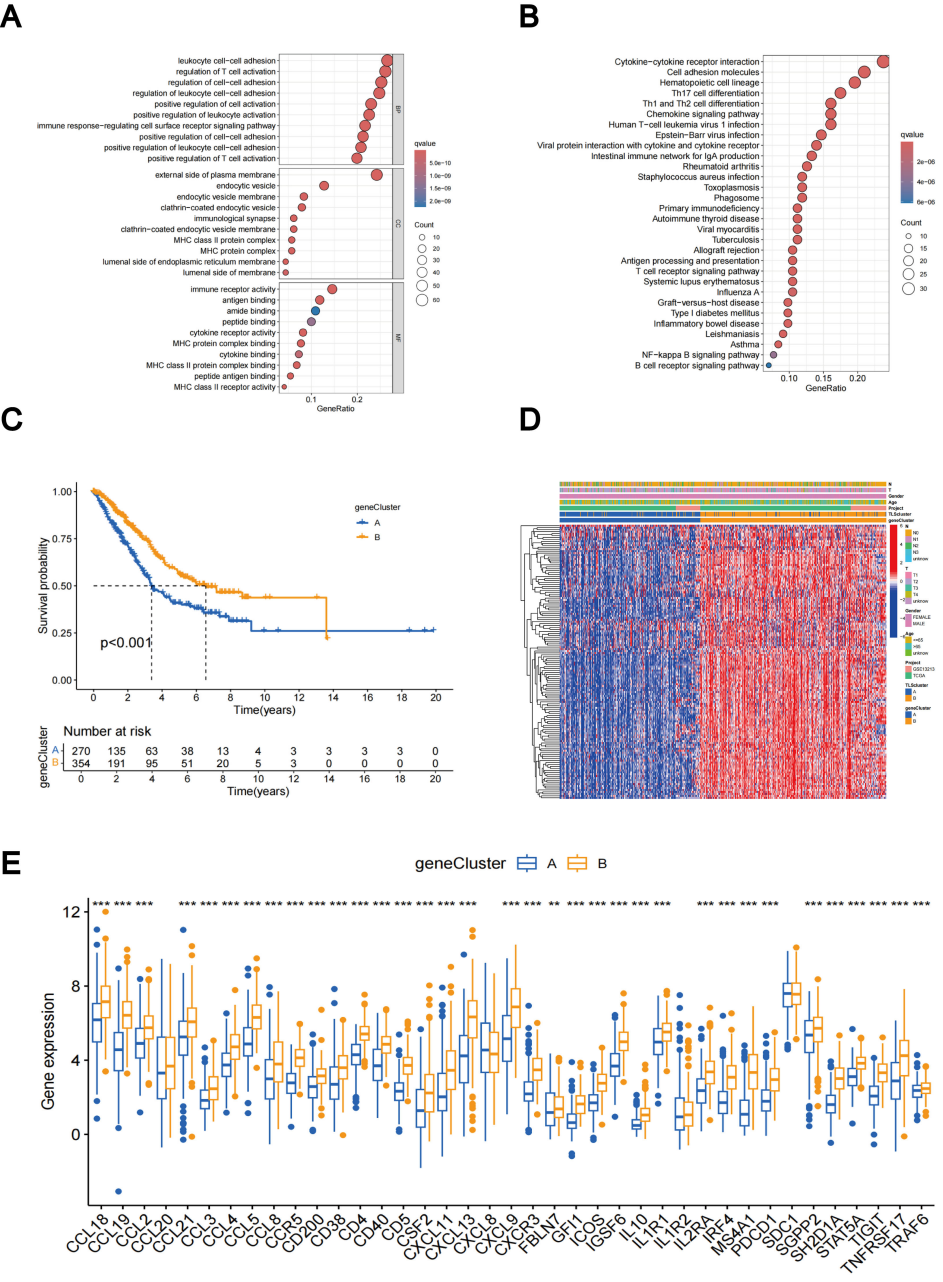
DEGs-based gene subtypes identification. **(A)** GO enrichment analyses of DEGs between different TLS subtypes. **(B)** KEGG enrichment analyses of DEGs between different TLS subtypes. **(C)** KM curves of gene subtypes. **(D)** Association between the two gene subtypes and clinicopathologic characteristics. **(E)** Differential expression of TLS-RGs across gene subtypes. **p < 0.01; ***p < 0.001.

Using the R package “limma”, we discovered 229 TLS subtype-related DEGs (**Supplementary Table 2**) and carried out functional enrichment analysis to explore the possible biological behavior associated with each TLS pattern (**Supplementary Figure 4A**). DEGs were predominantly enriched in immune process-related biological functions, indicating that TLS subtype gene-mediated immune process modifications are essential for LUAD regulation (**Figures 5A, B**). Furthermore, the involvement of TLS subtype-related genes in LUAD was explored using a consensus clustering approach to divide the patients into distinct gene subgroups based on the expression levels of TLS subtype-related genes. The findings indicated that The best grouping outcomes were achieved when patients were divided into two subgroups (**Supplementary Figures 4B–D**). Gene cluster B had a superior OS compared to gene cluster A, according to the KM curves, which showed a significant difference in TLS between the two gene clusters (p < 0.001; **Figure 5C**). **Figure 5D** shows an expression heat map of the genes associated with the two TLS subtypes. It was evident that these two gene clusters had different levels of gene expression (**Figure 5D**). Most TLS-RGs showed different expression levels between the two gene clusters (**Figure 5E**).

### Construction and certification of TLS scores

**Figure 6:**
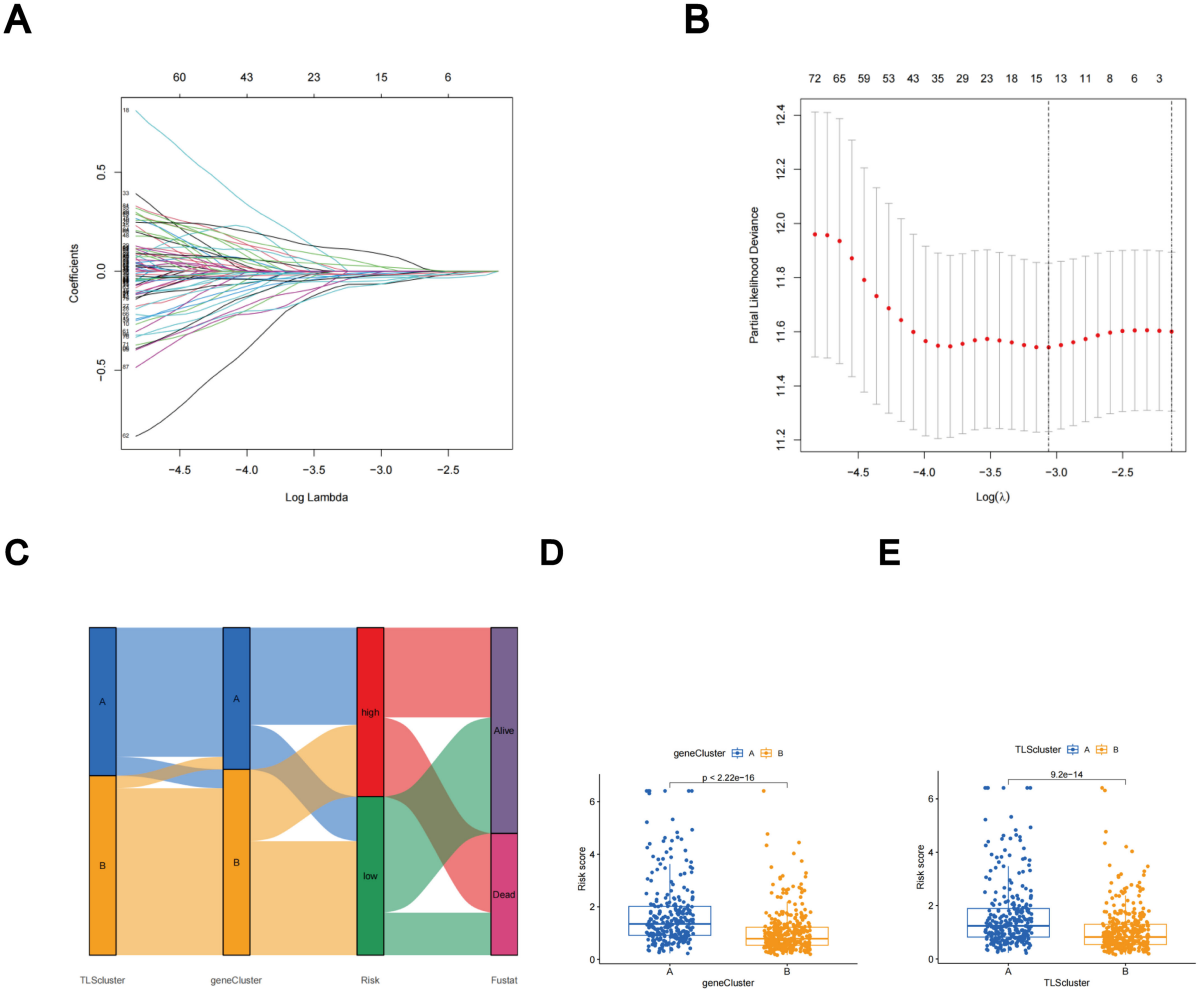
The LASSO regression and the construction of the TLS scores. **(A)** LASSO regression. **(B)** Profiles of LASSO coefficients. **(C)** Alluvial diagram illustrating the distribution of subtypes between groups based on survival outcomes and TLS scores. **(D)** Variations in TLS scores between gene subtypes. **(E)** Variations in TLS scores between TLS subtypes.

Subtype-related DEGs were used to establish the TLS scores. Initially, the LUAD patients were randomly divided into two groups: the training group and the test group, each consisting of 312 patients. The prognostic model was constructed using data from the training group, and the accuracy of the model was validated using the testing group. To determine the ideal prognostic signature for TLS subtype-related prognostic DEGs, LASSO and multivariate Cox analyses were performed. Fifteen OS-associated genes were identified using LASSO regression analysis, as shown by minimal partial likelihood deviance (**Figures 6A, B**). Subsequently, we evaluated these fifteen OS-associated genes using multivariate Cox regression analysis, leading to the identification of seven genes (CIITA, FCRL2, GBP1, BIRC3, SCGB1A1, CLDN18, and S100P). Based on the outcomes of multivariate cox regression analysis, the TLS score was computed using the following formula: Risk score = (-0.241448343 × expression of CIITA) + (-0.300276561 × expression of FCRL2 + (0.194538624 × expression of GBP1) + (0.174440808 × expression of BIRC3) + (-0.074347915 × expression of SCGB1A1) + (-0.081694457 × expression of CLDN18) + (0.058453204 × expression of S100P). Patients were then stratified into two risk groups, high and low, based on the median value of the risk scores.

**Figure 7:**
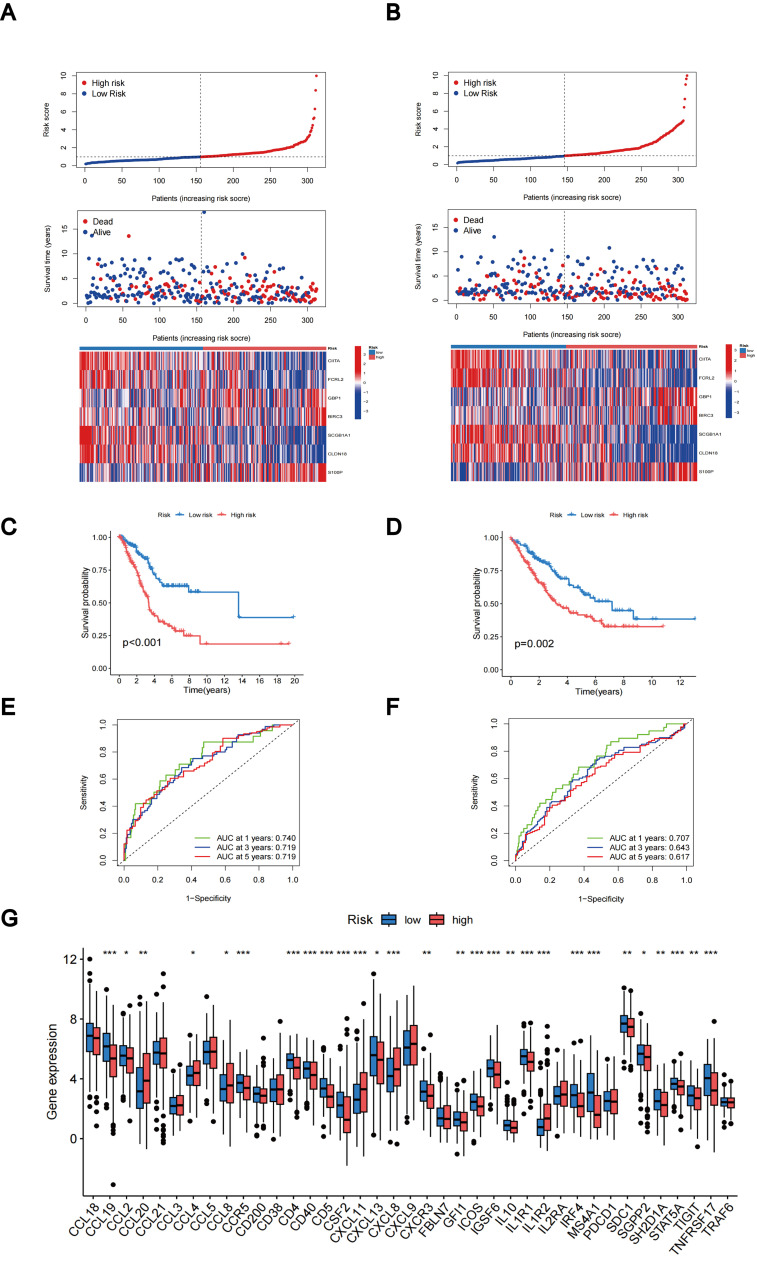
Evaluation and categorization outcomes of TLS scores. **(A)** The distribution of risk scores, survival status, and expression levels of seven prognostic genes in the training group. **(B)** The distribution of risk scores, survival status, and expression of seven prognostic genes in the testing group. **(C)** KM curve outcomes for LUAD patients with various TLS scores in the training group. **(D)** KM curve outcomes for LUAD patients with various TLS scores in the testing group. **(E)** Based on the TLS scores, ROC curves are used to estimate the sensitivity and specificity in the training group. **(F)** Using the TLS scores, ROC curves estimate the sensitivity and specificity in the testing group. **(G)** Variations in the expression of TLS-RGs in patients with different TLS scores. *p < 0.05; **p < 0.01; ***p < 0.001.

The distributions of patients among the two TLS subtypes, two gene subtypes, and two TLS score groups are shown in **Figure 6C**. The TLS scores exhibited variation in both the TLS and gene clusters, as shown in **Figures 6D, E**. TLS scores were lower in the TLS cluster B and gene cluster B. As TLS scores increased, the risk distribution plot illustrated a decrease in survival times and an increase in recurrence rates (**Figure 7A**). According to the KM survival curves, it was noted that patients with low scores exhibited superior OS compared to those with high scores (p < 0.001; **Figure 7C**). Furthermore, as depicted in **Figure 7E**, the AUC values for the 1-year, 3-year, and 5-year survival rates based on the TLS scores are 0.740, 0.719, and 0.719, respectively.

We calculated the TLS scores across the testing group to validate their prognostic performance. Based on the formula applied within the training group, we further categorized the patients into groups denoting low-risk and high-risk statuses. The relationship between TLS scores, survival times, and recurrence rates is depicted in the risk distribution plot (**Figure 7B**). Survival analysis unveiled a markedly superior prognosis within the low-risk group compared to the high-risk group (p = 0.002; **Figure 7D**). ROC curves in the testing group revealed that the TLS scores maintained relatively high AUC values (**Figure 7F**). In addition, we investigated the differential expression of TLS-RGs across various TLS scores. According to the results, among the 39 TLS-RGs, 28 genes exhibited differential expression, with the majority demonstrating high expression levels in the low-risk group (**Figure 7G**).

### Construction of a nomogram

**Figure 8:**
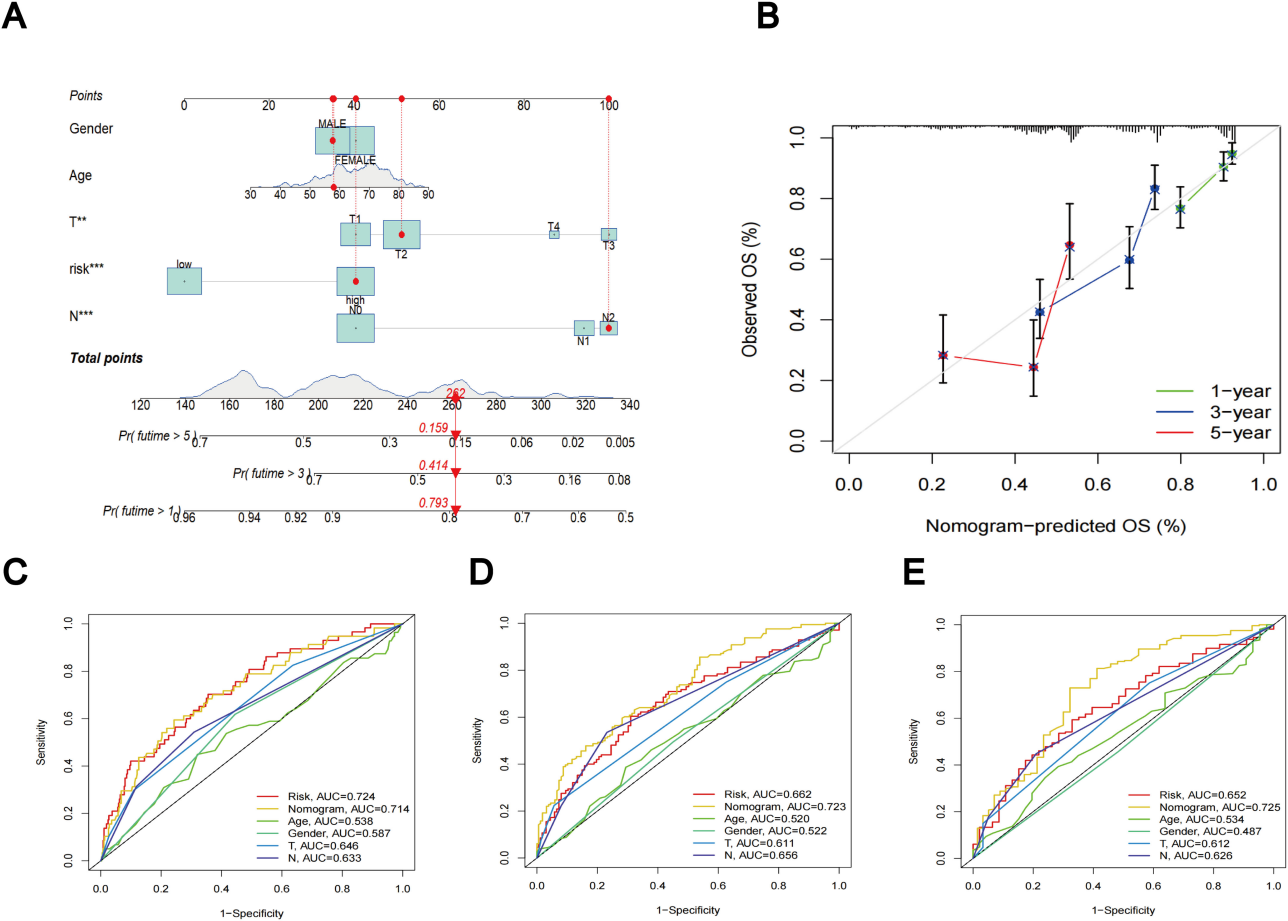
Nomogram results based on TLS scores and clinical factors. **(A)** Prognostic nomogram that predicts OS of LUAD patients, incorporating clinical features and TLS scores. **(B)** Prognostic nomogram Concordance Index findings. **(C)** ROC curves for nomogram, risk score, and clinical characteristics at 1 year. **(D)** ROC curves for nomogram, risk score, and clinical characteristics at 3 year. **(E)** ROC curves for nomogram, risk score, and clinical characteristics at 5 year.

Utilizing clinical features and TLS scores, we created a prognostic nomogram aimed at precisely predicting the prognosis of patients diagnosed with LUAD (**Figure 8A**). The results of the Concordance Index indicate the favorable predictive capability of the nomogram (**Figure 8B**). **Figures 8C–E** present the ROC curves and corresponding AUC values for risk score, nomogram, age, gender, T-stage, and N-stage at 1, 3, and 5 years, respectively. In **Figure 8C**, the risk score (AUC = 0.724) and nomogram (AUC = 0.714) demonstrate superior performance in predicting patient prognosis. **Figures 8D, E** reveal that the AUC values for the nomogram are 0.723 and 0.725, respectively, outperforming the risk score, which highlights its robust predictive capability.

### Evaluation of TME between different TLS scores

**Figure 9:**
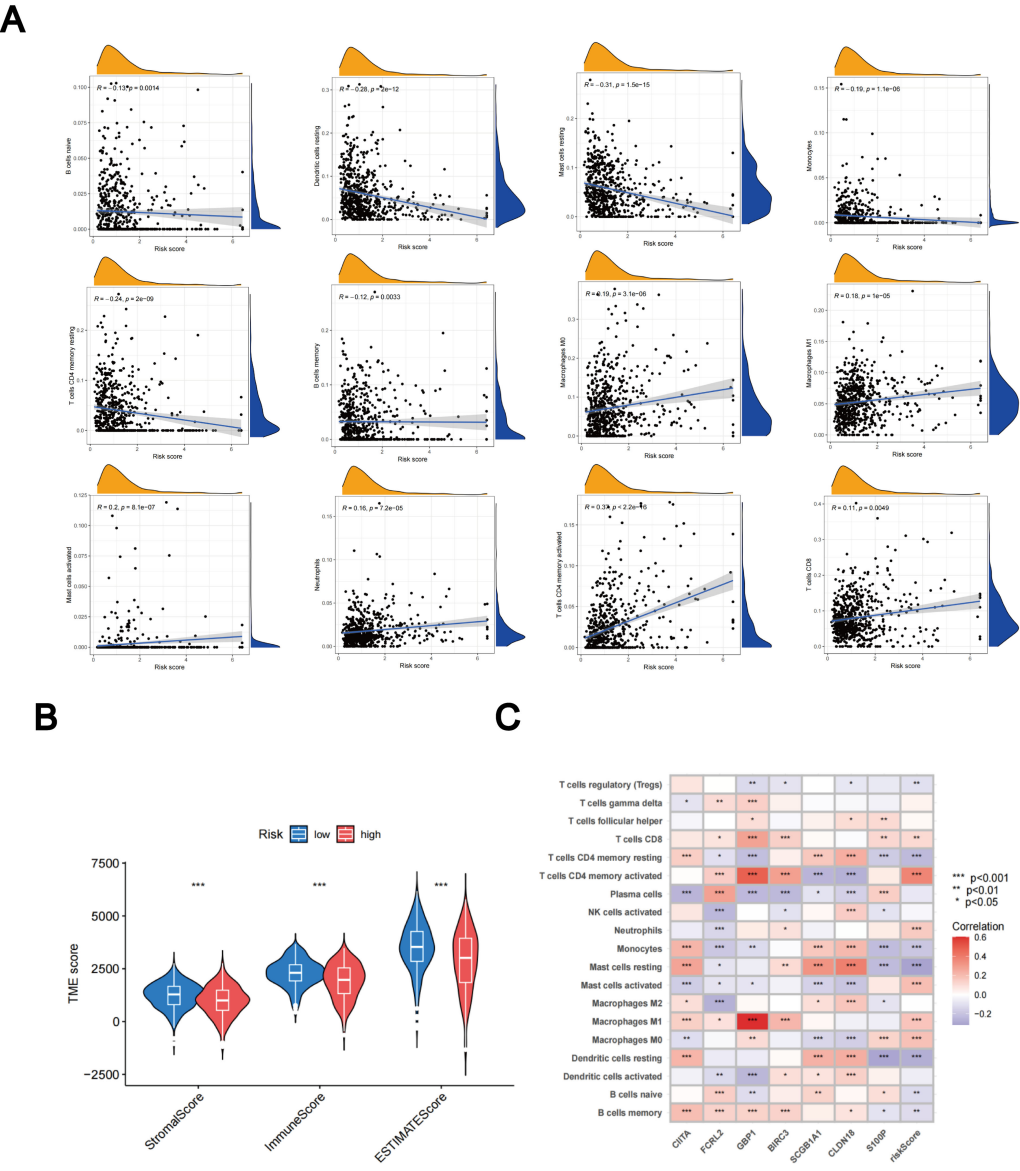
Assessment of the TME. **(A)** Relationships between immune cell types and TLS scores. **(B)** Associations between stromal scores, immune scores, and TLS scores. **(C)** Relationships between immune cell abundance and the expression of seven genes. *p < 0.05; **p < 0.01; ***p < 0.001.

Using the CIBERSORT algorithm, we evaluated the relationship between the TLS scores and the abundance of immune cells. The scatter diagrams indicate a positive correlation between TLS scores and M0 macrophages, M1 macrophages, neutrophils, CD8 + T cells, activated memory CD4 + T cells, and activated mast cells. Conversely, they show a negative correlation with naive B cells, memory B cells, resting mast cells, resting dendritic cells, monocytes, and resting memory CD4 + T cells (**Figure 9A**). High stromal and immune scores were strongly correlated with low TLS scores (**Figure 9B**). Furthermore, we observed a significant correlation between the expression of these seven genes and the majority of immune cells (**Figure 9C**).

### Relationship of TLS scores with TMB and CSC index

**Figure 10:**
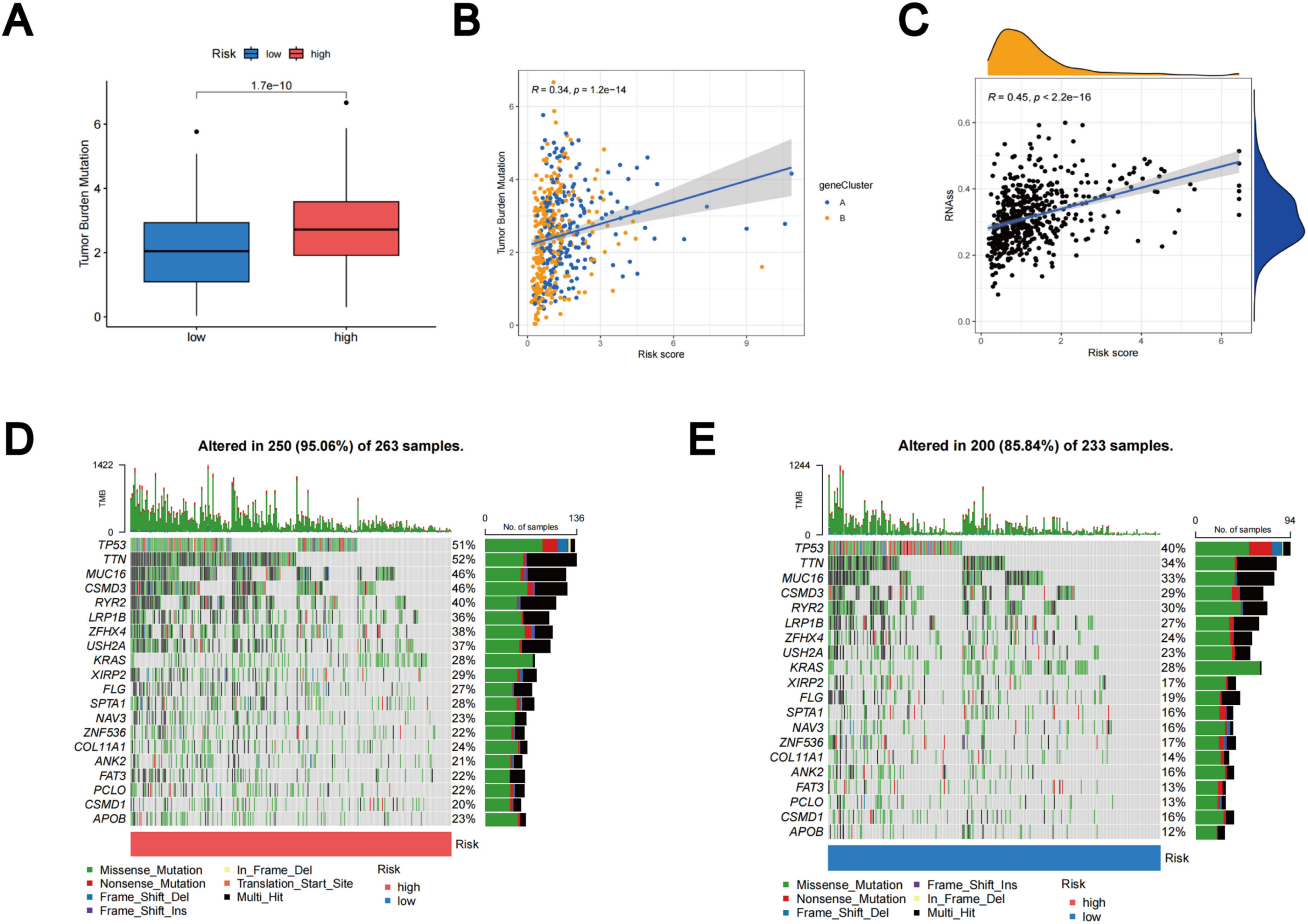
Outcomes of TMB, CSC, and tumor mutation landscape analysis. **(A)** The TMB expression in different TLS scores. **(B)** Spearman correlation analysis between TMB and TLS scores. **(C)** Relationships between the CSC index and TLS scores. **(D)** Somatic mutation features resulting in high TLS scores. **(E)** Somatic mutation features resulting in low TLS scores. Every column denoted a distinct patient. TMB was displayed in the top bar plot.

Based on accumulating evidence, patients with high TMB may potentially benefit from immunotherapy due to their increased neoantigen counts (45, 46). Our analysis of mutation data from the TCGA LUAD cohort indicates that individuals in the high-risk group may benefit from immunotherapy, as the high subgroup displays higher TMB (**Figure 10A**). Furthermore, Spearmanor correlation analysis revealed a positive correlation between TMB and TLS scores (R = 0.34, p < 0.001), as illustrated in **Figure 10B**. The linear association between the CSC index and TLS scores is depicted in **Figure 10C**. Our data analysis revealed a positive correlation between CSC and TLS scores (R = 0.45, p < 0.001). These findings imply that LUAD cells exhibiting higher TLS scores tend to exhibit enhanced stem cell characteristics and diminished levels of cellular differentiation (**Figure 10C**). Subsequently, we delved into the differences in somatic mutation distribution between the two TLS scoring groups within the TCGA-LUAD cohort. The top ten mutated genes were identified as TP53, TTN, MUC16, CSMD3, RYR2, LRP1B, ZFHX4, USH2A, KRAS, and XIRP2, respectively (**Figures 10D, E**). Notably, the frequency of mutations observed in patients with high TLS scores was significantly higher. These findings further emphasize the potential clinical significance of TLS scores in individuals with LUAD, providing crucial insights for tailored treatment strategies and prognostic assessments.

### Drug sensitivity analysis in different TLS scores

**Figure 11:**
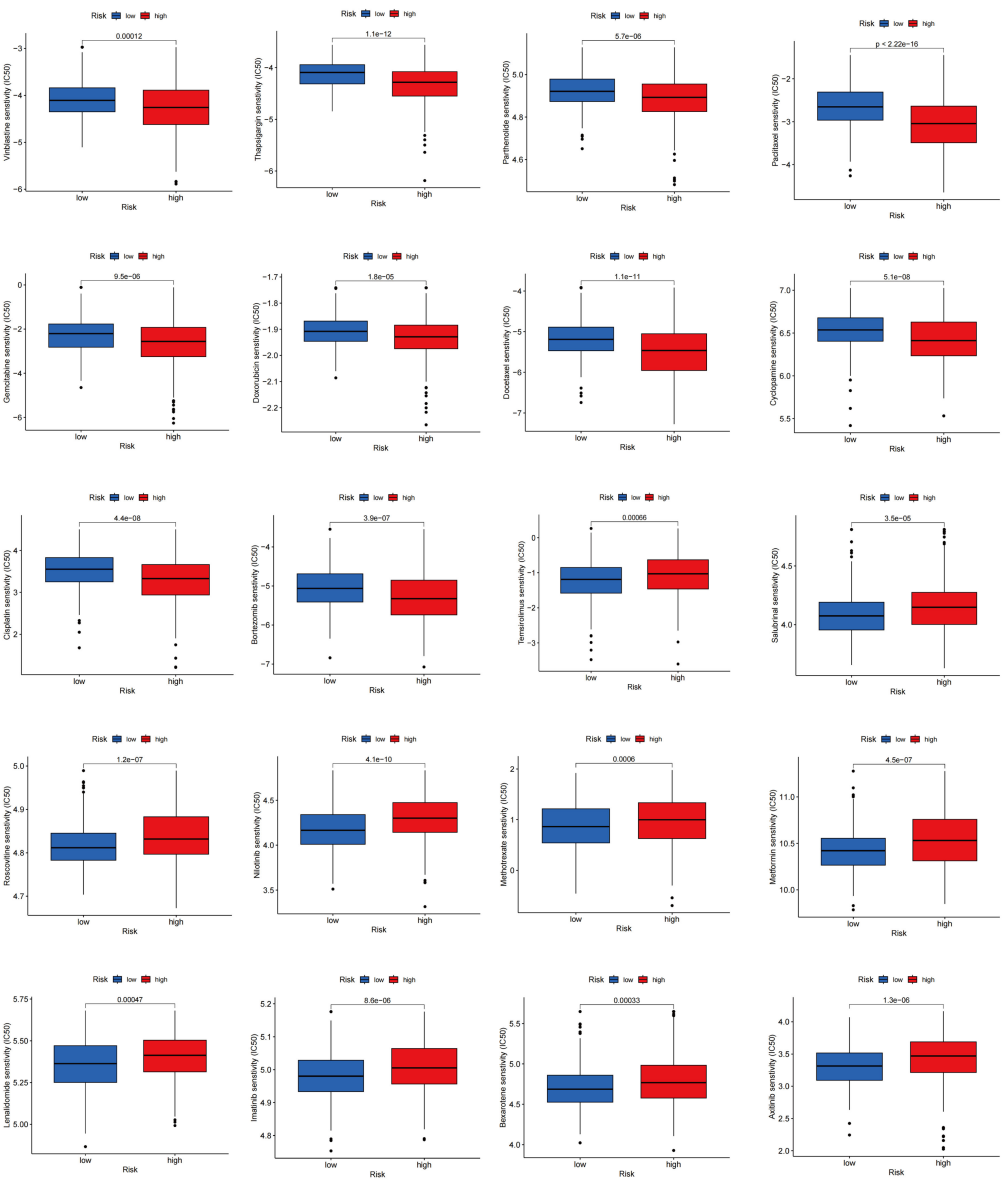
Relationships between drug sensitivity and different TLS score groups.

Drug sensitivity reflects the reaction of a patient to drug therapy. To evaluate the sensitivity of patients to various medications commonly used in LUAD treatment, we selected several drugs for evaluation. Interestingly, patients with high TLS scores showed lower IC50 values for vinblastine, thapsigargin, parthenolide, paclitaxel, gemcitabine, doxorubicin, docetaxel, cyclopamine, cisplatin, and bortezomib, whereas those with low TLS scores showed significantly lower IC50 values for therapeutics such as temsirolimus, salubrinal, roscovitine, nilotinib, methotrexate, metformin, lenalidomide, lmatinib, bexarotene, and axitinib. Overall, these findings underscore the relationship between TLS-RGs and drug sensitivity, indicating potential implications for therapeutic outcomes (**Figure 11**).

### Single-cell sequencing analysis

**Figure 12:**
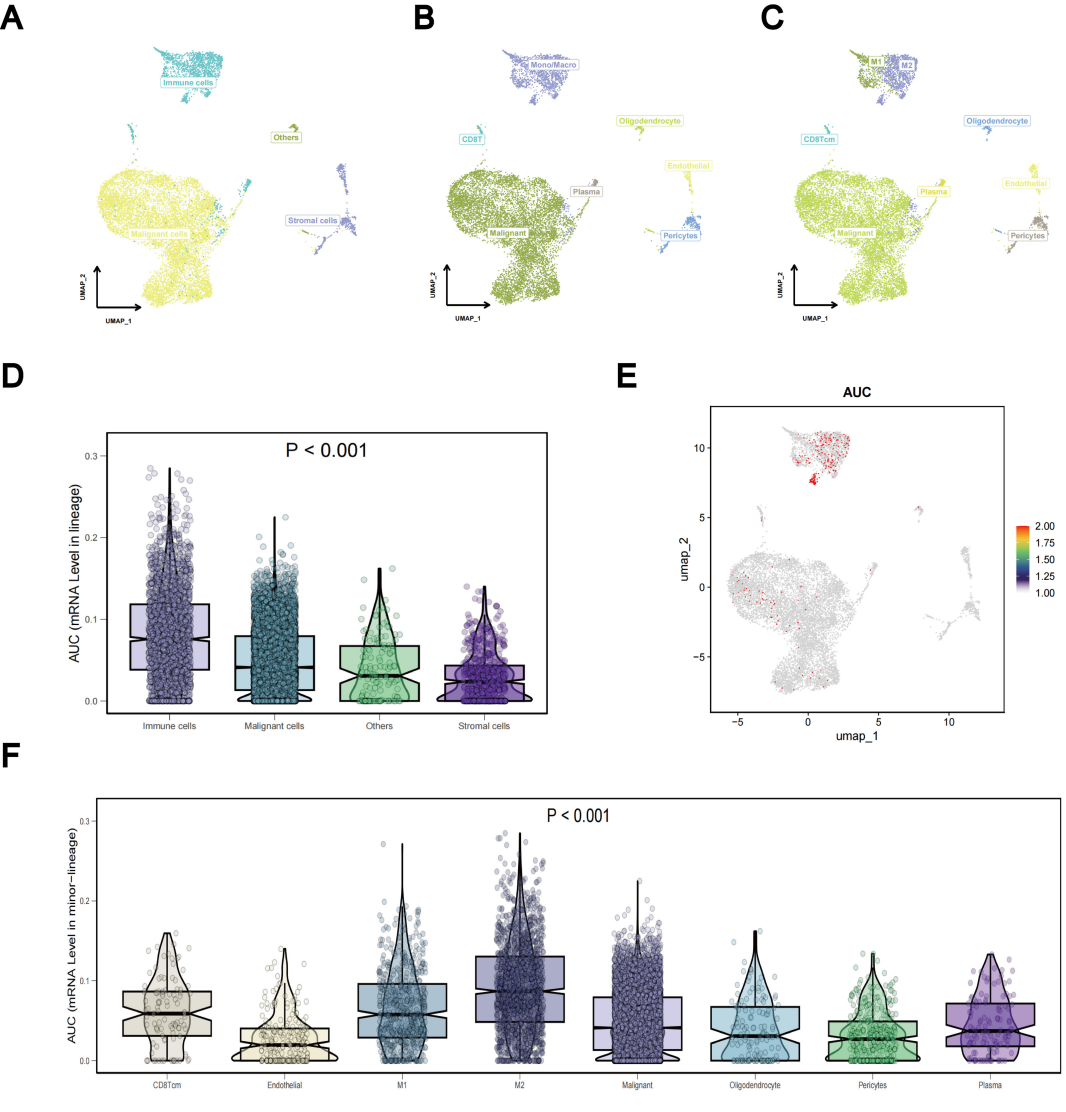
Single-cell sequencing analysis of TLS-RGs in GSE143423. **(A–C)** Aggregation of consolidated data in the UMAP. **(D)** Differences in TLS-RGs expression between cells. **(E)** Single-cell TLS-RGs AUCell scoring. **(F)** Differences in TLS-RGs expression between specific cells.

To investigate differences in TLS gene expression among different LUAD cell types, we systematically analyzed LUAD single-cell sequencing data from three datasets: GSE143423, GSE146100, and GSE153935. For each dataset, integration involved implementing batch correlation techniques, followed by dimensionality reduction methods and subsequent unsupervised clustering procedures. In the resultant graph of the UMAP analysis of the dataset GSE143423, it was clear that different cell populations were distinguished based on their expression profiles, including malignant, immune, and stromal cells (**Figure 12A**). Notably, mononuclear macrophages constituted the primary immune cell population, with a predominance of M2 over M1 cells within the macrophage subset (**Figures 12B, C**). Using the Kruskal-Wallis rank sum test, we assessed the differential expression of the TLS gene set using AUCell scoring in different cell types. Our findings revealed that the TLS gene set exhibited differential expression in immune, stromal, and malignant cells, with TLS-RGs showing notably high expression levels in immune and malignant cells (p < 0.001; **Figures 12D–F**). Similar results were observed in gene sets GSE146100 and GSE153935 (**Supplementary Figures 5**, **6**).

### TLS-RGs validation with LUAD cells and tissues

**Figure 13:**
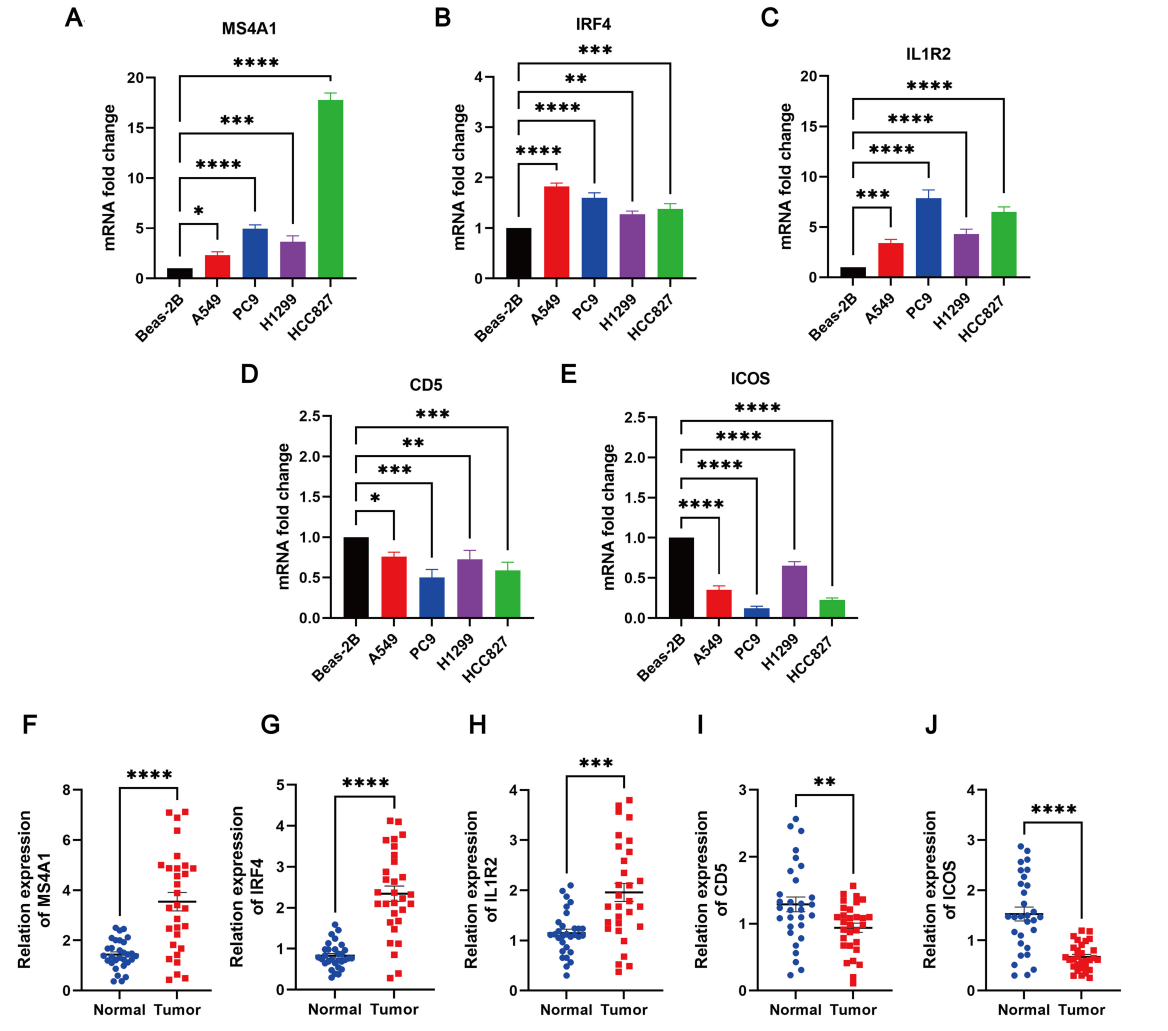
The degree of 5 TLS prognostic signature mRNA expression by qRT-PCR. The mRNA expression levels of **(A)** MS4A1; **(B)** IRF4; **(C)** IL1R2; **(D)** CD5; **(E)** ICOS in Beas-2B, PC9, A549, H1299 and HCC827 cell lines; **(F–J)** Relative expression of MS4A1, IRF4, IL1R2, CD5, and ICOS in normal and LUAD tissues by qRT-PCR. *p < 0.05; **p < 0.01; ***p < 0.001; ****p < 0.0001.

The expression levels of MS4A1, IRF4, IL1R2, CD5, and ICOS in LUAD cell lines (PC9, A549, H1299, and HCC827) and Beas-2B control cell line were evaluated using qRT-PCR. The results indicated that MS4A1, IRF4, and IL1R2 were upregulated in LUAD cell lines, while CD5 and ICOS were downregulated (**Figures 13A–E**). Additionally, the expression levels of these five TLS prognosis-related genes were assessed in 30 pairs of LUAD tissues and their adjacent normal tissues. It was found that MS4A1, IRF4, and IL1R2 were significantly upregulated in LUAD tissues, whereas CD5 and ICOS were downregulated (**Figures 13F–J**). Immunohistochemical images of lung cancer from the HPA database corroborated these findings (**Supplementary Figure 7**), which were also consistent with the results from the TCGA cohort.

## Discussion

Lung cancer stands as one of the leading causes of cancer-related fatalities worldwide (47), with LUAD emerging as the predominant subtype. It accounts for over one million deaths annually across the globe (48, 49). Therefore, additional methods for guiding the treatment of LUAD are urgently required. TLS has gradually become an important factor associated with prognosis and carcinogenesis (50, 51). Therefore, through an in-depth exploration of the function of TLS in LUAD, we aim to gain a better understanding of its impact on patient prognosis and offer more precise and effective strategies for LUAD treatment. This will contribute to the development of personalized treatment plans and provide the best treatment options for each patient. Ultimately, this approach seeks to reduce discrepancies in prognosis, enhance treatment outcomes, and improve survival rates.

The findings of our study demonstrate widespread changes in TLS-RGs in LUAD at both the transcriptional and genomic levels. Utilizing 39 TLS-RGs, we stratified LUAD into two distinct TLS subtypes. Patients with TLS subtype A showed lower OS and more advanced clinicopathological characteristics than those with TLS subtype B. Significantly, there existed a notable contrast in the infiltration levels of most immune cells between the two TLS subtypes, with subtype B exhibiting heightened levels of 20 immune cell types compared to subtype A. In TLS subtype B, we also observed significant differences in immune activation, including natural killer cell-mediated cytotoxicity, activation of chemokine signaling pathways, T-cell and B-cell receptor signaling pathways, cytokine receptor interactions, as well as Toll-like and Jak-Stat receptor signaling pathways. These findings indicate that TLS-RGs play a critical predictive role in assessing the response to LUAD immunotherapy and determining clinical prognosis, thus exerting a significant influence on patient treatment and recovery.

Utilizing the DEGs identified in TLS subtypes, we stratified LUAD patients into two gene subtypes. Notably, the OS of gene subtype B surpassed that of gene subtype A. Subsequently, prognostic models were constructed, and risk scores were calculated. LUAD patients were classified into two distinct groups: high risk and low risk. There were notable differences in clinicopathological features, mutations, prognosis, CSC index, TMB, and medication responsiveness between patients with different TLS scores. Compared to those with high TLS scores, patients exhibiting low TLS scores demonstrated markedly longer survival times and lower rates of recurrence. The somatic mutation frequency was notably higher in patients with high TLS scores compared to those with low scores, potentially indicating a poorer prognosis. It is well known that cancer patients with CSC have a poor prognosis (52). Several studies have reported a negative association between TMB and the prognosis of tumor patients (53–55). Both CSC and TMB show a positive correlation with TLS scores, indicating a worse prognosis for patients with high TLS scores. Patients with low TLS scores exhibited markedly lower IC50 values for temsirolimus, salubrinal, roscovitine, nilotinib, methotrexate, metformin, lenalidomide, lmatinib, bexarotene, and axitinib. Conversely, patients with high TLS scores exhibited significantly lower IC50 values for vinblastine, thapsigargin, parthenolide, paclitaxel, gemcitabine, doxorubicin, docetaxel, cyclopamine, cisplatin, and bortezomib, suggesting that individuals with varying TLS scores respond differently to medication. These findings imply that TLS scores have the potential to personalize treatment strategies for patients with LUAD. Additionally, by integrating TLS scores with tumor stage, we developed a quantitative nomogram, which not only enhanced performance but also facilitated the effective utilization of TLS scores.

The significant role of the TME in cancer development and drug resistance is widely recognized (56–58). The primary cellular constituents of the TME are innate immune cells, including tumor-associated neutrophils, tumor-associated macrophages, tumor-associated dendritic cells, and adaptive immune cells, such as regulatory T cells (59). Significant disparities were identified between the two molecular subtypes concerning TLS scores, TME, and the relative abundance of 19 TIICs. T-cells play a pivotal role in cancer immunotherapy (60–62). TLS subtype B, characterized by a low TLS score and associated with better prognosis, exhibited heightened infiltration of activated CD4+, CD8+ T cells, and gamma delta T cells. Emerging evidence indicates that B cells also contribute to the immune response against tumors (63–65). In our study, we observed that the numbers of activated B cells, activated CD8+ T cells, and immature B cells were significantly higher in TLS subtype B and subtypes with lower TLS scores compared to TLS subtype A. Additionally, we conducted single-cell sequencing analysis to examine the distribution of TLS-RGs within cells. Lastly, we validated the expression of five genes related to prognosis through qRT-PCR analysis. In the LUAD cell lines PC9, A549, H1299, and HCC827, elevated expression levels of MS4A1, IRF4, and IL1R2 were observed compared to the Beas-2B control cell line. These observations align with data from the TCGA cohort, suggesting that these genes may serve as pivotal markers for LUAD. Conversely, we noted significantly lower expression levels of CD5 and ICOS in LUAD cell lines. These distinct expression profiles underscore the intricate interplay between tumor cells and the immune system in LUAD. Our findings underscore the potential of these genes as prognostic biomarkers and therapeutic targets in LUAD.

Despite the thorough analyses conducted in this study, several limitations must be acknowledged. First, all samples were obtained retrospectively, and all analyses were limited to data from publicly available databases, which may have introduced inherent biases in case selection, potentially affecting the results. Second, larger prospective studies and *in vitro* and *in vivo* experiments are necessary to thoroughly validate these findings. Third, the lack of clinical validation limits the direct applicability of the results in real-world clinical settings. Additionally, the use of multiple datasets may have resulted in batch effects, despite efforts at normalization. These limitations indicate that further studies, including larger cohort studies and extensive experimental validation, are necessary to confirm these findings.

## Conclusion

In conclusion, our extensive exploration of TLS-RGs in LUAD unveiled their promising role as biomarkers for prognostic prediction in patients with this disease. We found that TLS-RGs exert a notable influence on the immunological landscape of LUAD patients, providing valuable predictive information for both immunotherapy and chemotherapy outcomes. The results of this study highlighted the significant clinical consequences of TLS-RGs and offered novel insights into the development of tailored immunotherapeutic approaches for patients with LUAD.

## Data Availability

The original contributions presented in the study are included in the article/**Supplementary Material**. Further inquiries can be directed to the corresponding authors.
