# Supplementary material for: Multi-omics profiling and experimental verification of tertiary lymphoid structure-related genes: molecular subgroups, immune infiltration, and prognostic implications in lung adenocarcinoma
Source: Front Immunol. 2024 Sep 19;15:1453220. doi: 10.3389/fimmu.2024.1453220 (PMC11446812; doi:10.3389/fimmu.2024.1453220)
Supplement: Supplementary file 1 [file DataSheet1.docx]

Supplementary Figures


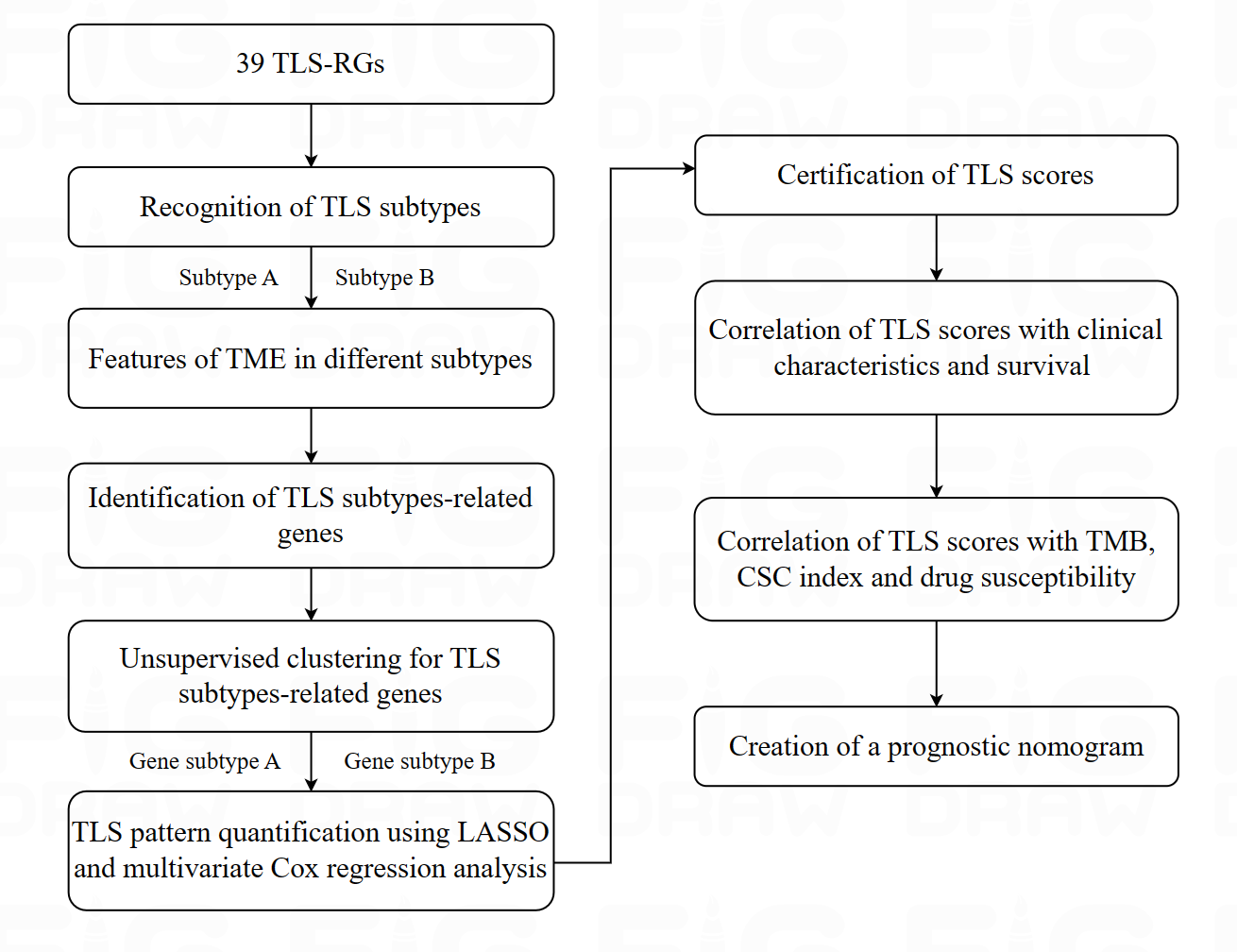


Supplementary Figure 1. The complete analytical procedure of the study.


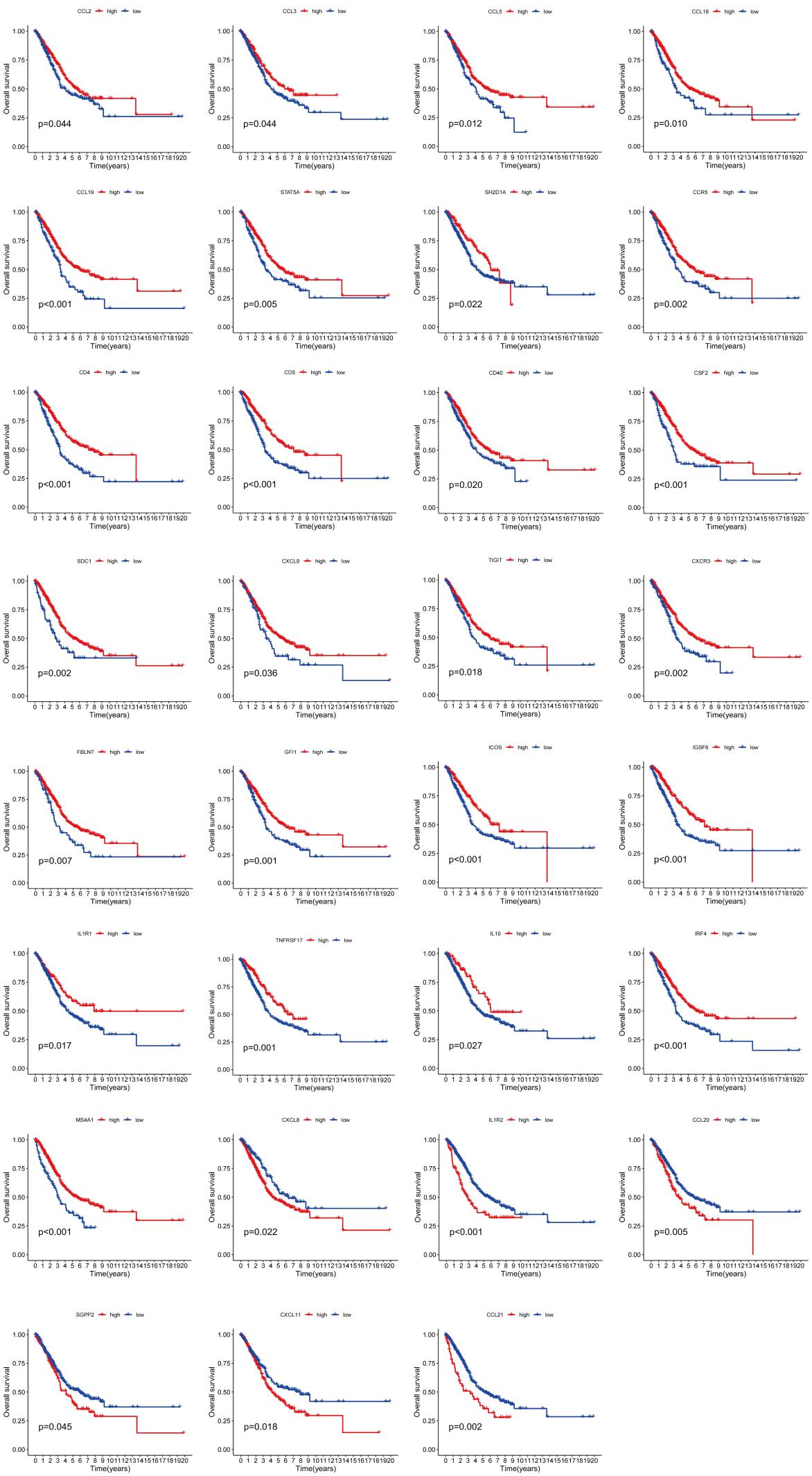


Supplementary Figure 2. KM curve results of LUAD patients with different TLS-RGs expression.


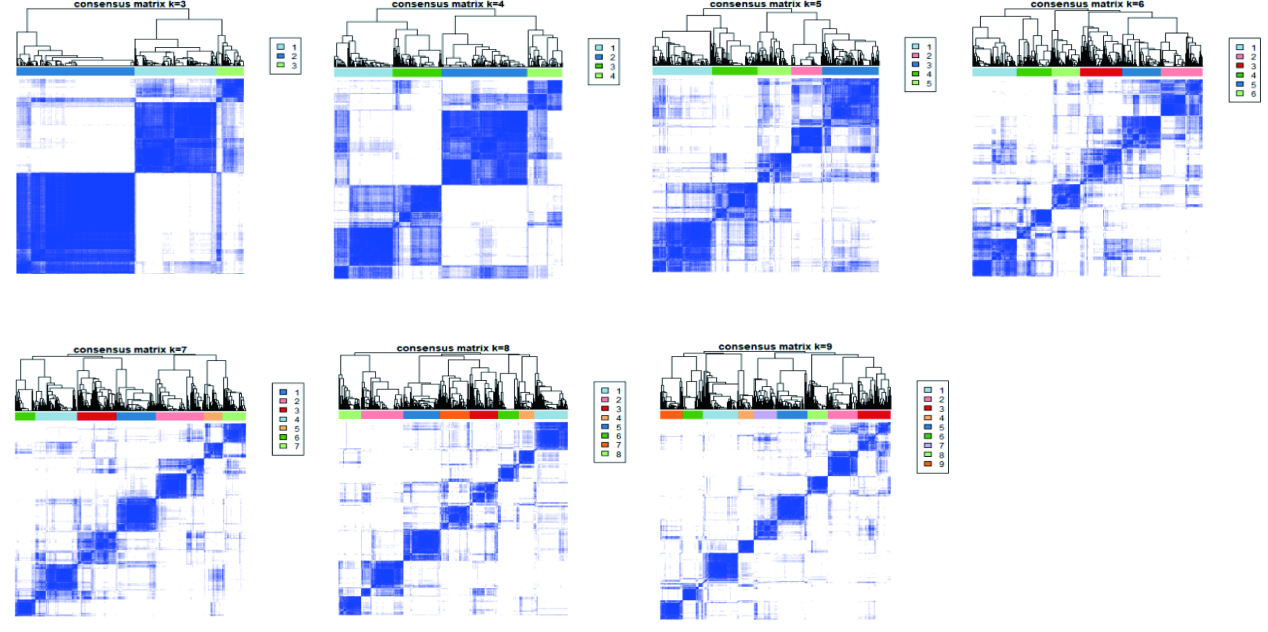
Supplementary Figure 3. Consensus matrix heatmaps and unsupervised clustering of genes associated with TLS for k = 3–9.


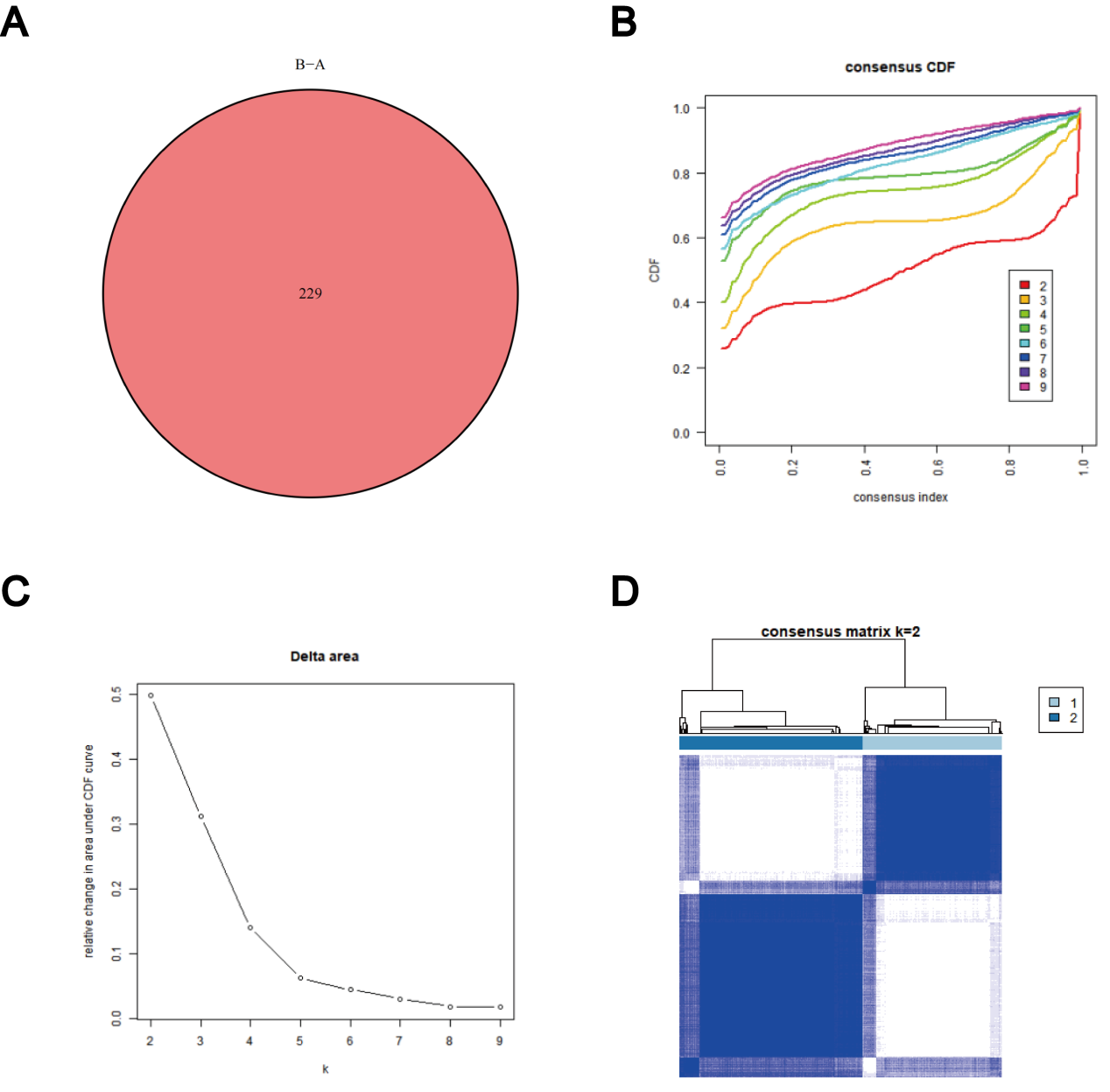
Supplementary Figure 4. Two TLS subtypes in the LUAD cohort were identified as gene subtypes based on DEGs. **(A)** 229 TLS subtype-related DEGs. **(B)** For each tested k, the CDF displays the cumulative fraction of every sample co-clustering at the specified consensus index (1.0 = co-clustered 100% of the time). **(C)** The consensus clustering delta area curve, which shows how the area under the CDF curve changes relative to k − 1 for each category number k. The vertical axis shows the relative change in the area under the CDF curve, while the horizontal axis displays the category number k. **(D)** Heatmap of the consensus matrix indicating the correlation area and two clusters (k = 2).


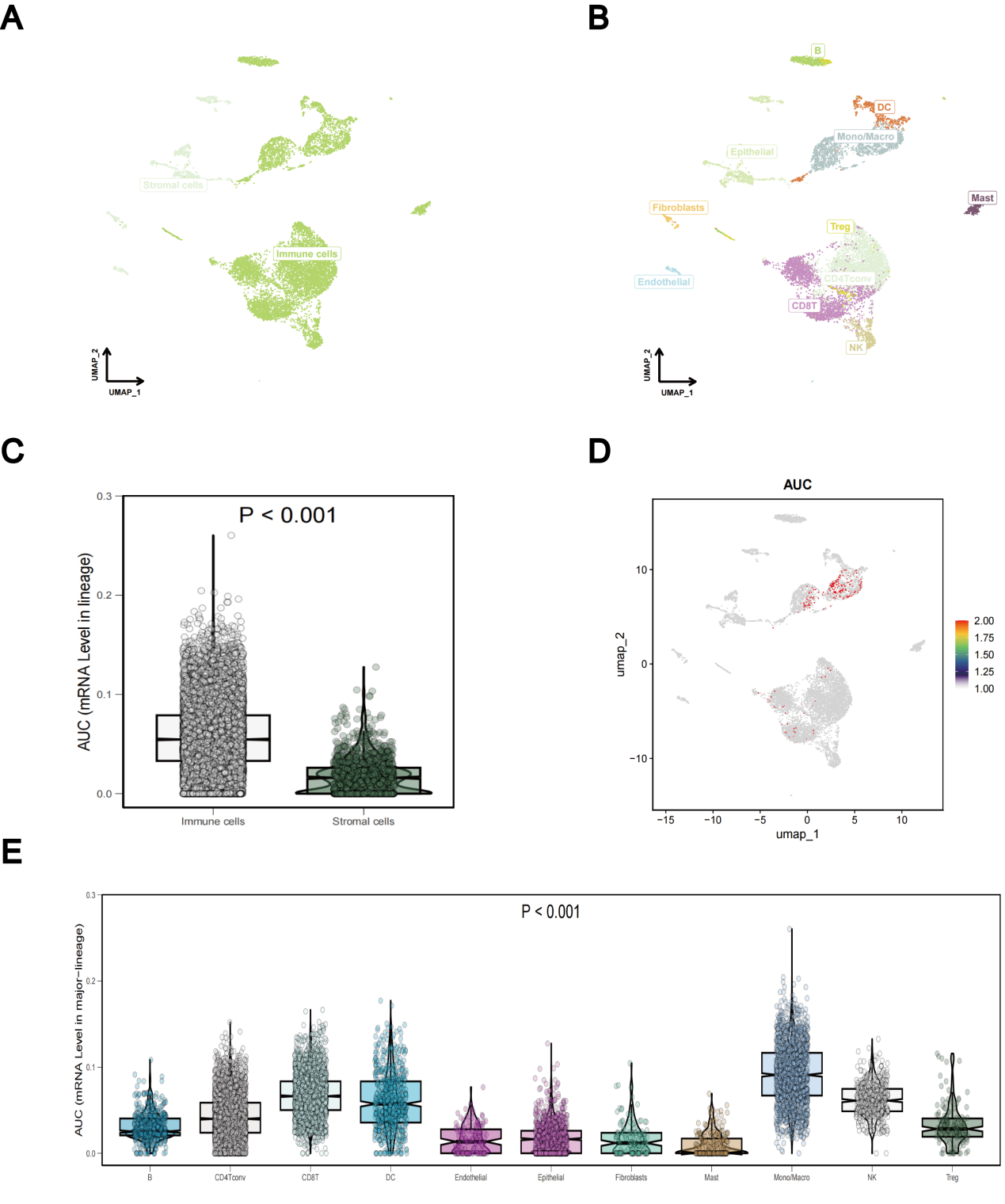


Supplementary Figure 5. Single-cell sequencing analysis of the TLS-RGs in GSE146100. **(A-B)** Aggregation of consolidated data in the UMAP. **(C)** Differences in TLS-RGs expression between cells. **(D)** Single-cell TLS-RGs AUCell scoring. **(E)** Differences in TLS-RGs expression between specific cells.


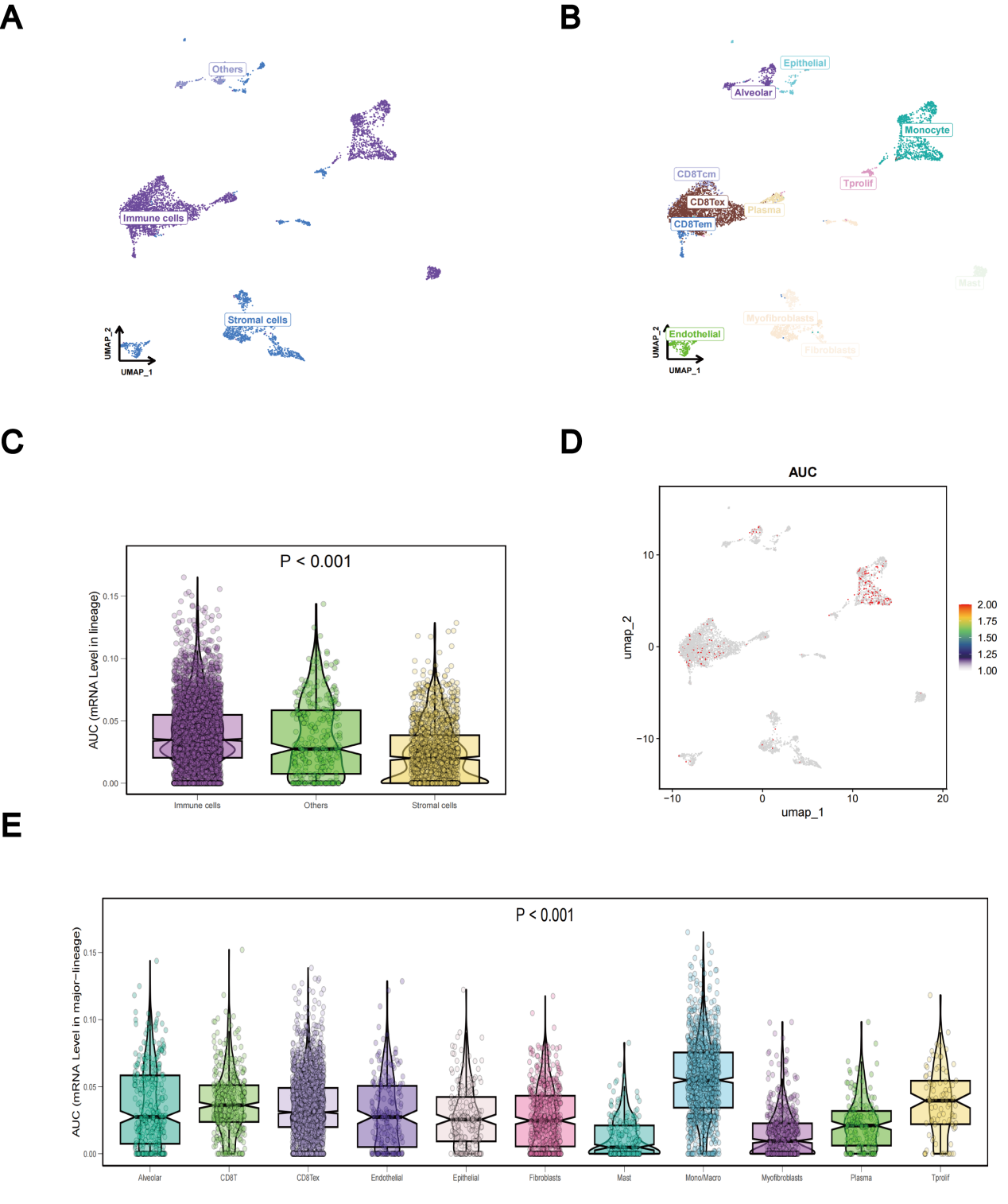
Supplementary Figure 6. Single-cell sequencing analysis of the TLS-RGs in GSE153935. **(A-B)** Aggregation of consolidated data in the UMAP. **(C)** Differences in TLS-RGs expression between cells. **(D)** Single-cell TLS-RGs AUCell scoring. **(E)** Differences in TLS-RGs expression between specific cells.


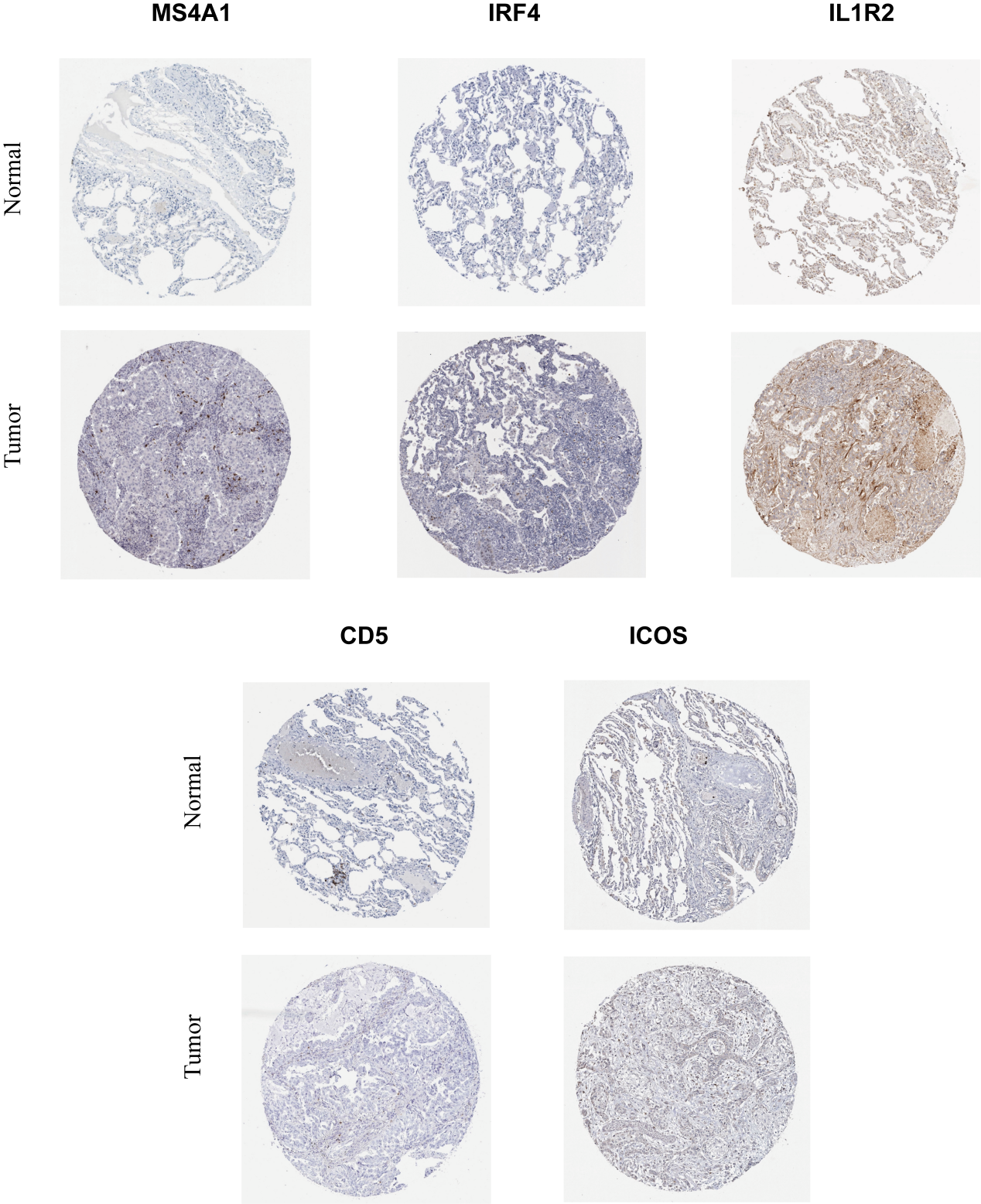


Supplementary Figure 7. Immunohistochemical images of 5 TLS prognostic genes in normal and lung cancer tissues from the HPA database.
